# Breastmilk microbiota and its association with infant iron deficiency anaemia: A 16S rRNA metagenomic study in Peruvian mothers cohort

**DOI:** 10.1371/journal.pone.0352428

**Published:** 2026-08-27

**Authors:** Arturo Octavio Gonzales-Rodriguez, Luis Edgardo Gonzales-Huerta, Paolo Alberto Wong Chero, Luz Estela Vera-Silva, Guillermo Uceda-Campos, Francesca Antonia Reale

**Affiliations:** 1 Faculty of Human Medicine, University of Piura, Lima, Peru; 2 Department of Biochemistry, Chemistry Institute, Universidade de São Paulo, São Paulo, Brazil; 3 Ente Nazionale per l'Energia Elettrica, Lima, Peru; All India Institute of Medical Sciences, INDIA

## Abstract

Anaemia is one of the most important public health challenges in developing countries. The global burden is estimated at 1.8 billion people, affecting approximately 30% of all children. Despite the implementation of multiple strategies over several decades, up to 42.5% of children under 3 years of age suffer anaemia in rural areas of Peru. We studied the microbiota of breastmilk (hBM) from mothers with children under the age of 1 and analysed its association with their iron deficiency anaemia.

46 mother-child pairs were recruited from Talara, a town on the northern coast of Peru and classified according to the clinical status of the children. Children with anaemia were also tested for ferritin level to classify them as iron deficiency anaemia (IDA) or non-iron deficiency anaemia (non-IDA). hBM samples were taken from the mothers after careful instruction to reduce the risk of sample contamination and the microbiome was analyzed using 16S rRNA sequencing.

*Streptococcus* and *Staphylococcus* were the predominant genera, with 90% of bacterial abundance being explained by 14 genera. Alpha diversity analysis showed that hBM from mothers of children with non-IDA had higher levels of bacterial richness than hBM from healthy (p < 0.01) and IDA (p < 0.05) participants. Principal coordinates analysis did not yield differential clusters but showed a disparity in the spread of samples for non-IDA group when compared with the other groups. IDA group showed higher burden of *Corynebacterium*, *Acinetobacter*, *Paludibacter* and *Nitrospira*, while exhibiting a trend towards a lower burden of *Streptococcus*. No statistically significant differences were identified on demographic characteristics.

This study suggests that hBM microbiota may differ in mothers of children with IDA and non-IDA, highlighting the necessity of further in-depth research to elucidate potential factors associated with its pathogenesis.

## Introduction

Anaemia is one of the most important public health problems [1]. According to the world health organization (WHO) the global burden is 1.8 billion people, affecting approximately 30% of all children under 5 years of age [2]. Anaemia is mainly caused by iron deficiency, the most common nutritional deficiency in the world [3]. Children that suffer from anaemia within their first 1000 days of life have a significantly higher risk of developing irreversible developmental, cognitive, and behavioural disorders [1,4].

For several decades, initiatives to reduce the burden of anaemia in Peruvian population have been unsuccessful. In 2017, the national 4-year plan to reduce anaemia from 46% to 19% was implemented [5]. However, the 2023 National Demographic and Health Survey reported that 42.5% of children in rural areas and one third of children nationwide (33.4%) between 6 and 35 months of age have anaemia [6]. Specifically, in the northern coastal region of Piura, anaemia affected 39.7% of children aged 6–35 months in 2023 [6]. Further analysis of Peruvian population, revealed that socioeconomic inequalities based on household- and community-related factors contribute significantly to the development of anaemia in children under 5 years, although it emphasized heterogeneity across different regions in Peru, with Piura emerging as one of the most affected by socioeconomic disparities [7].

Exclusive breastfeeding during the first six months of life is strongly recommended by the WHO [1]. Evidence indicates that breastfeeding reduces infant mortality (WHO, 2013) and promotes overall health and well-being [8]. Beyond its nutritional value, human breastmilk (hBM) microbiota inhibits the growth of pathogenic bacteria and provides a wide range of microorganisms that contribute to the establishment of the infant gut microbiome during the first three months of life [9]. Studies of hBM from healthy mothers have identified Enterobacteriaceae, Streptococcaceae and Staphylococcaceae as predominant taxa in the early postnatal period, while hBM also provides oligosaccharides that favour the growth of beneficial gut bacteria such as Bifidobacteriaceae and Lactobacillaceae [10–12].

*Bifidobacterium* spp. not only inhibits the growth of pathogenic bacteria, but also contributes to the degradation and uptake of human milk oligosaccharides (HMOs) through cross-feeding mechanisms in which HMO-degrading strains release metabolic byproducts that can be used by other strains unable to directly utilize these substrates, promoting cooperative interactions within the microbial community [13]. Furthermore, Short-Chain Fatty Acids (SCFAs) synthesised by the gut microbiota acidify the intestinal lumen, increasing iron solubility and promoting its reduction to the ferrous form, thereby enhancing intestinal absorption [14,15]. In contrast, disruption of gut microbiota colonisation can severely impair epithelial homeostasis and contribute to disease development [16,17]. Other genera such as *Veillonella* and *Streptococcus* have also been associated with SCFA production, beneficial anti-inflammatory properties, and improved insulin sensitivity [18].

Despite the well-recognised benefits of breastfeeding, the impact of hBM microbiota on iron absorption—and its potential contribution to iron deficiency anaemia (IDA)—is still poorly understood. To distinguish microbiota patterns potentially associated with iron deficiency from those associated with anaemia more broadly, children with non-iron deficiency anaemia (non-IDA) and healthy children were included as comparison groups. Therefore, the aim of this study was to investigate potential associations between hBM microbiota and iron deficiency anaemia (IDA).

## Materials and methods

### Population

Forty-six mother-child pairs were recruited in Talara, Piura, a city on the northern coast of Peru. Recruitment was conducted from October 4 to December 20, 2019. Inclusion criteria comprised children between 3 and 12 months of age, who were at the time being breastfed at least four times daily, and whose mothers were between 18 and 40 years old. Exclusion criteria included children with low birth weight, recent antibiotic use by the mother (within the last 15 days), mothers undergoing chemotherapy, those with HIV infection, those who had delivered by caesarean section, and those presenting with mastitis or other inflammatory signs of the mammary gland.

Breastmilk (hBM) was collected, and groups were categorized according to the child’s anaemia status, yielding 15 mother-child pairs with iron-deficiency anaemia (IDA), 15 with anaemia without iron deficiency (non-IDA), and 16 without anaemia. The non-IDA and healthy groups were included as comparison groups to facilitate the identification of microbiota characteristics potentially associated with iron deficiency rather than anaemia status alone. Population homogeneity was determined by assessing the following variables: child’s diet, parasitic infections, prenatal health history, and socio-demographic status.

### Demographics

Socio-economic status was assessed with a modified survey based on the National Demographic and Family Health Survey (ENDES), which assesses households conditions and organizes results to determine socio-economic development from a geographical perspective. We applied a modified survey based on the Food and Agriculture Organization of the United Nations (FAO) nutritional survey corresponding to the age bracket of infants [19]. In addition, information on place of residence, gestational duration, and number of children was recorded.

Anthropometric data were obtained from the official Ministry of Health registry (“Healthy Child Checkup Card”) included in the participants’ medical records. Information recorded at birth included weight and supine length, as well as infant age and sex.

### Ethics

All participants provided written informed consent prior to inclusion in the study. In the case of infants, consent was obtained from one of the parents or legal guardians. The study was approved by the Ethics and Research Committee at the Faculty of Medicine of Universidad San Martin de Porres, Lima, Peru.

### Laboratory

Faecal samples from infants were collected and preserved in 10% formaldehyde at room temperature exclusively for parasitological analysis. Samples were examined seven days after collection. Microscopic evaluation was performed using both direct smear and sedimentation techniques, following the methodology described by Tello [20]. Haemoglobin levels were measured in both mothers and their infants using a portable hemoglobinometer (HemoCue® Hb 201 + , Cat. No. 121707, Ängelholm, Sweden). This method was selected for practical reasons, as anaemia status was measured directly in the field during community-based visits**.** Study groups were established following the World Health Organization’s (WHO) guidelines for diagnosis of anaemia in infants [21]. Ferritin levels were assessed in children that fulfilled the diagnostic criteria for anaemia according to the WHO [21]. Quantification of ferritin was performed with Ferrozine method (Wiener Lab®, Cat. N° 1410057, Rosario, Argentina).

### Collection of breastmilk

Between 5 and 50 mL of breast milk were collected from each mother recruited into the study. Sampling took place between 09:00 and 14:00 hours. No dietary restrictions were imposed prior to collection; participants were only instructed to abstain from breastfeeding for at least two hours before sample collection. Following confirmation of compliance, the nipples and surrounding skin were cleaned using sterile cotton swabs and saline. An equivalent volume of milk was manually expressed from each breast, discarding the initial drops to minimise potential contamination with skin-colonising bacteria. Immediately after collection, samples were refrigerated at 4 °C and kept under these conditions for a maximum of 2 hours. They were then stored at –20 °C for no longer than 48 hours before being transferred to –80 °C for long-term preservation. All hBM samples were maintained at –80 °C until analysis.

### DNA extraction

3 mL of hBM were centrifuged at 13,000 g for 10 min. The top cream layer was cleared using a sterile cotton swab and the supernatant discarded. We used the PureLink™ Microbiome DNA Purification commercial kit (Invitrogen™, Cat. No. A29790, Thermo Fisher Scientific, Waltham, MA, USA) for metagenomic DNA extraction, according to manufacturer´s instructions. The final extraction volume was 50 µL. DNA quantification was performed with a Qubit™ dsDNA BR Assay.

### Sequencing analysis

16S sequencing was performed in 46 hBM samples, aiming for the amplification of V3 and V4 hypervariable regions of the 16S ribosomal RNA. Equimolar concentrations of primers were used [22,23].

Amplicons of approximately 460 bp, including overhang sequences, were generated. Amplification reactions were prepared with 5 ng/µL of microbial DNA, 1 µM of primer, and 12.5 µL of 2 × KAPA HiFi HotStart ReadyMix. PCR conditions were set at: initial denaturation for 5 min at 95 °C, followed by 30 cycles of 15 s at 95 °C, 15 s at 57 °C, and 30 s at 72 °C, with a final extension for 5 min at 72 °C. Amplicons were purified from free and dimerized primers using the Agencourt AMPure XP system. A second PCR was then performed to incorporate sequencing adapters, enabling library normalization and pooling. Sequencing was carried out on a MiSeq Illumina platform (Illumina. 16S Metagenomic Sequencing Library Preparation. Part #15044223 Rev. B, 2013).

### Bioinformatics analysis of bacterial composition and statistics

Sequences were clustered into Operational Taxonomic Units (OTUs), which represent groups of highly similar sequences, using a subsampled open-reference approach with a 97% sequence similarity threshold. Singleton OTUs were retained in the analysis. OTU selection was conducted with Kraken v2.0.1 metagenomic classification software. OTU identified as mitochondrial DNA, or chloroplasts were eliminated from analysis. Additionally, OTUs with fewer than 100 reads were filtered out to enhance data reliability, reducing the final counts to 89, 116, and 149 OTUs in the healthy, IDA, and non-IDA groups, respectively.

In healthy participants, sequencing generated an average of 209,199 reads, identifying microbial diversity across 229 families and 548 genera at 97% similarity, with OTU counts ranging from 195 to 548. In IDA participants, sequencing yielded an average of 215,767 reads, identifying 210 families and 479 genera, with OTU counts ranging from 238 and 479. In the non-IDA group, sequencing produced 182,037 reads identifying 276 families and 612 genera, with OTU counts ranging from 221 to 621. Across all groups, single-read and two-read OTUs represented approximately 42–46% of the total identified OTUs.

Rarefaction curves were analysed using alpha diversity indices, including Chao1, Shannon and Simpson indexes. Beta diversity was assessed using Aitchison distance calculated from appropriately transformed abundance data. Differences in microbial community composition among the healthy, IDA, and non-IDA groups were evaluated using permutational multivariate analysis of variance (PERMANOVA).

Statistical analysis was performed on R Studio for Windows v.4.4.2. Shapiro-Wilk test for assessing normal distribution was performed as required. Wilcoxon test, One-way ANOVA followed by Tukey’s HSD post-hoc tests and Kruskal-Wallis statistical tests were performed. A p-value < 0.05 was considered statistically significant.

## Results

### Demographics

**Fig 1:**
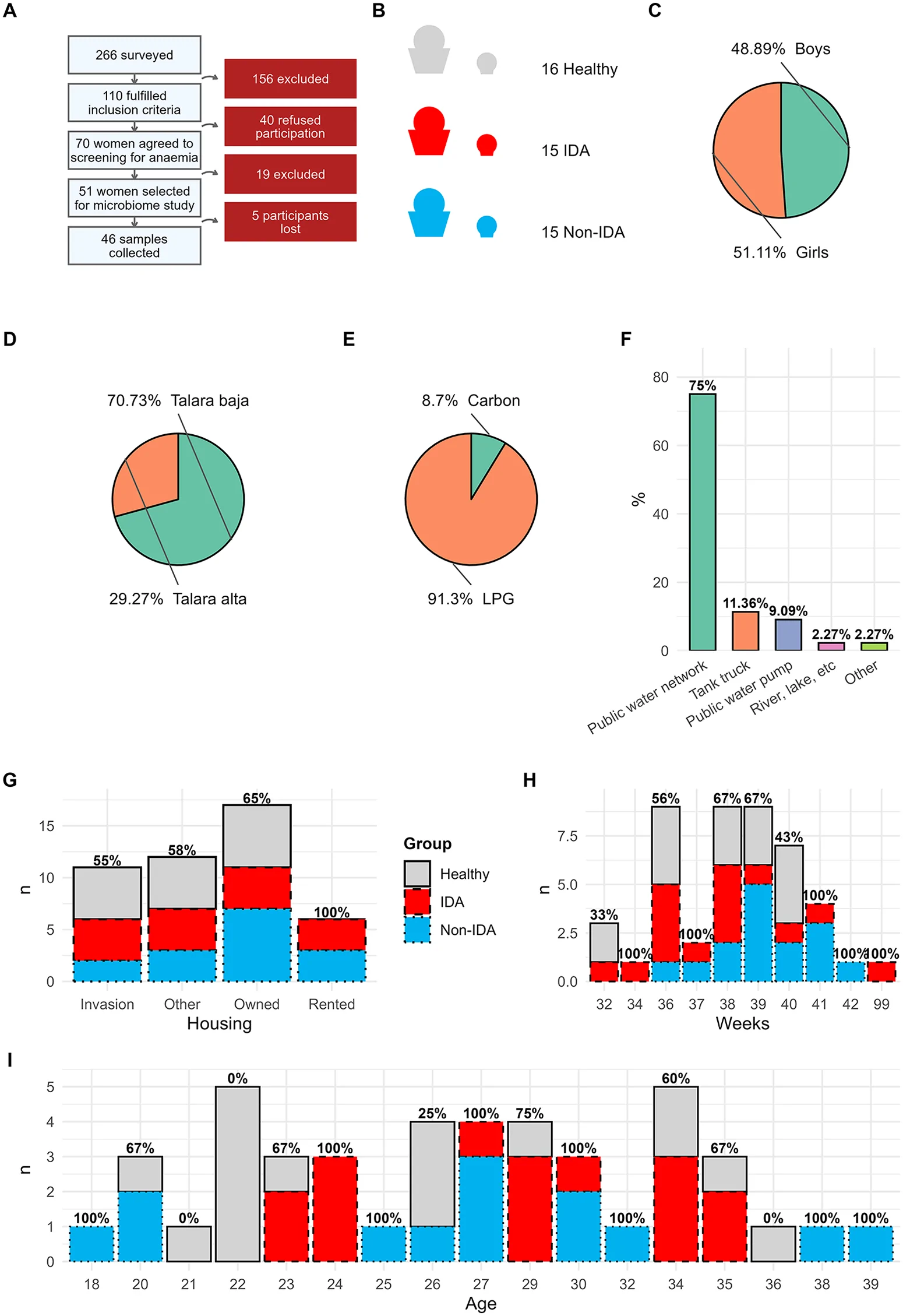
Flowchart of population selection and socio-demographics characteristics.

A total of 46 mother-child pairs were enrolled in the study and grouped according to the child’s anaemia status. 16 pairs were healthy, 15 had IDA and 15 had non-IDA (Fig 1). Among the participants, 70.7% resided in Talara Baja, 50% reported living in owner-occupied houses, predominantly built with bricks and cement, 71.7% had access to a public water supply and sewer network at home, and over 90% used liquefied petroleum gas (LPG) as the primary cooking fuel. None of these conditions were statistically associated with anaemia status.

Mothers’ ages ranged from 18 to 39 years, with a mean age of 27.6. They gave birth between 32 and 42 weeks of gestation, with a mean gestational age of 37.9 weeks. Neither maternal age nor gestational age at birth was significantly associated with the children’s anaemia status at the time of the study (Fig 1). The average number of children per mother was 2.73, ranging from 1 to 6. Maternal haemoglobin concentrations ranged from 12.9 ± 2.5 mg/dL, with no statistically significant differences observed between groups.

The baseline characteristics of the study population showed no statistically significant differences between groups, including children’s age, sex, gestational age, birth weight or birth length (Supplementary data, S1 Table). Children’s ages ranged from 3 to 12 months, with an average age of 7.7 months for the study population. 48.9% of children were males versus 51.1% of females. All children under the age of 6 months (n = 10) received hBM exclusively. Most mothers (n = 44) reported direct breastfeeding and 2 reported expressed breastfeeding in the non-IDA group. In children over 6 months, the dietary profile shows a low consumption of legumes across all groups and a preference for infant formulas and canned milk as a substitute for breast milk. Although Talara is a coastal city, fish consumption is low, with foods like liver and kidney being more prevalent, especially among children with anaemia. Egg consumption was highest in children with non-IDA (76.9%, p-value = 0.024), and yellow and orange vegetables were prominent due to their inclusion in common per at this stage. On average, children had 2.6 meals per day, with no statistically significant differences between groups (healthy, IDA, non-IDA).

Anaemia was diagnosed using the haemoglobin cut-offs defined by WHO for diagnosing anaemia [21]. Among the ten children aged 3–5 months, two were diagnosed with anaemia, with haemoglobin concentration below 9.5 g/dL. In the 6–12 months age group, fifteen children were identified with mild anaemia (< 10.5 g/dL), and nine with moderate anaemia (<9.5 g/dL). IDA was defined as ferritin concentrations below 12 μg/L. The mean Hb concentration among healthy participants was 10.9 g/dL for those under 6 months and 12.3 g/dL for those aged 6–12 months. In children with IDA, Hb mean concentration was 9.9 mg/dL, with mean ferritin concentration of 3.4 μg/L. Non-IDA group had a mean Hb concentration of 9.8 g/dL and a mean ferritin concentration of 61.1 μg/L.

In the parasitological analysis performed, *Blastocystis hominis* was the only parasite detected. Although this species is commonly reported in the northern coast of Peru [24], in our study its frequency was low across all groups (0 in healthy participants, 1 in the IDA group and 2 in the non-IDA group). In all positive cases, parasite counts did not exceed two trophozoites per microscopic field, suggesting no apparent clinical impact (Supplementary Data, S2 Table). Therefore, the parasitic burden is unlikely to have influenced anaemia status among the groups.

### Relative abundance of bacterial families and genera in hBM samples

**Fig 2:**
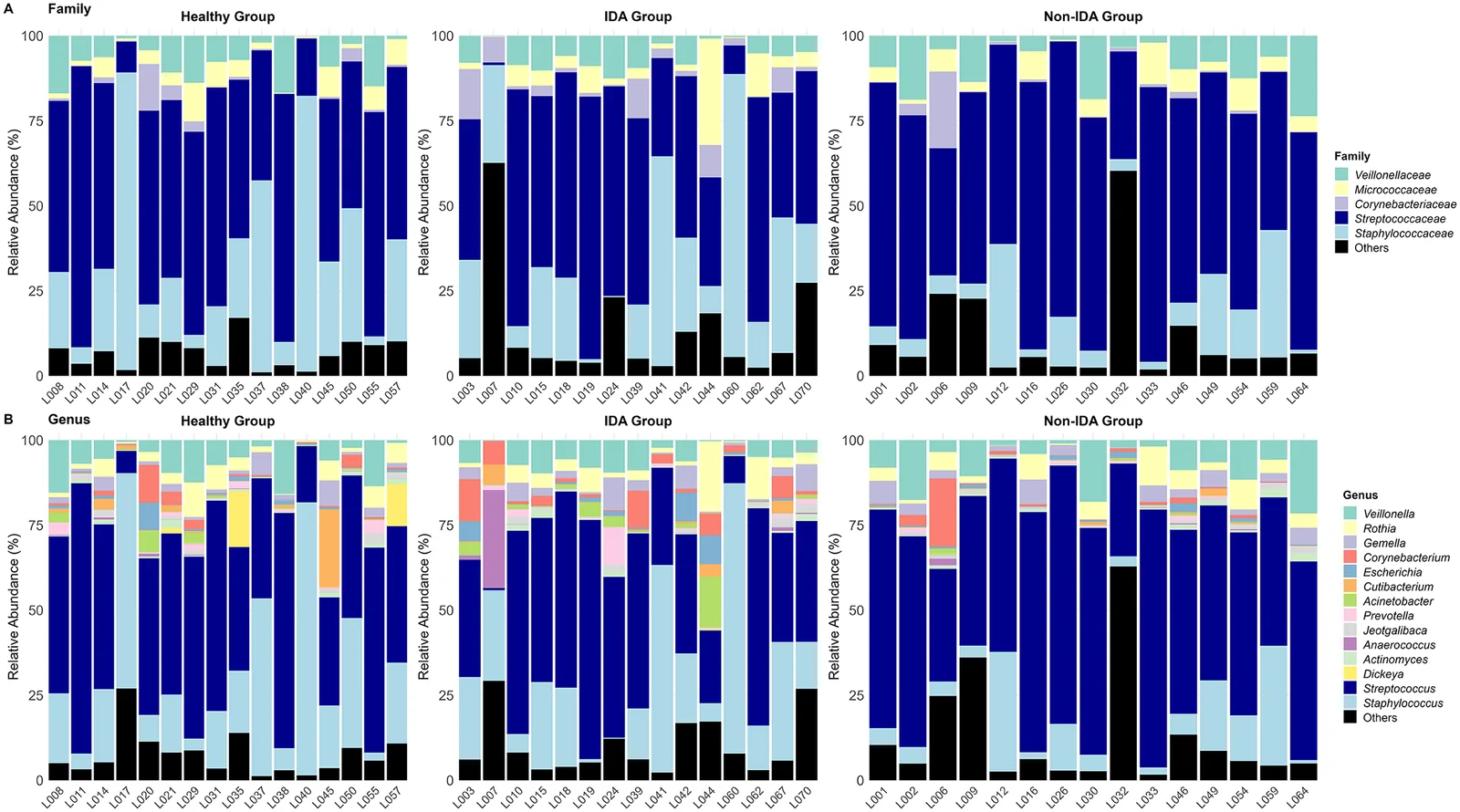
Relative abundance of bacterial families and genera.

We determined the most common bacterial families and genera across all hBM samples collected for the study (Supplementary data, S3 Fig). Streptococcaceae, Staphylococcaceae, Bacillales, Micrococcaceae and Veillonellaceae represented 90% of all families detected across all samples. Analysis at the genus level, identified that 14 genera comprised 90% of bacterial abundance in hBM samples, where most abundant genera across samples were *Streptococcus* and *Staphylococcus* (Fig 2).

### Microbial richness in hBM

Alpha diversity and richness in hBM were assessed using Chao 1, Shannon and Simpson indexes. Chao1 index, which estimates richness based on the number of rare species in a microbial population, mean values for healthy participants were 422.25 (SD ± 84.20) versus 436.07 (SD ± 80.00) and 539.73 (SD ± 137.77) for IDA and non-IDA groups respectively. Increased diversity was established for non-IDA participants in comparison with healthy participants (p-value = 0.045).

**Fig 3:**
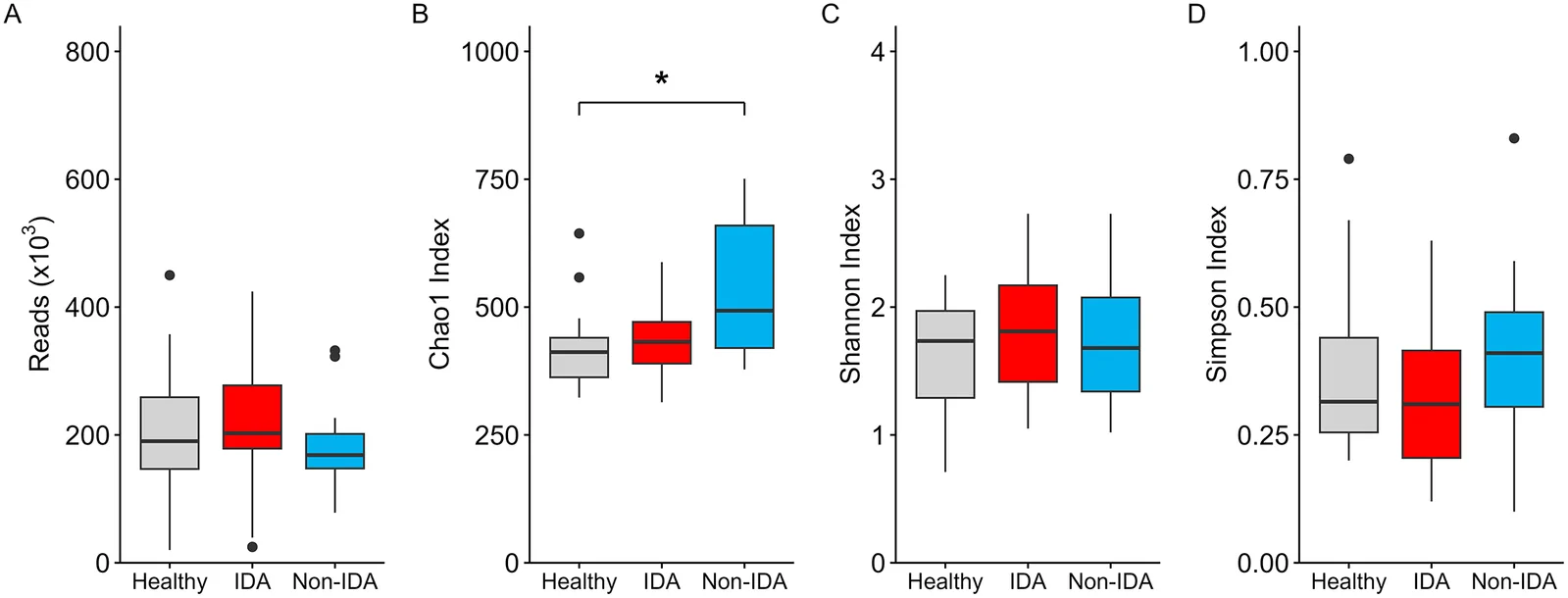
Boxplots for comparison of total reads and alpha diversity tests.

Regarding the Shannon index, a high degree of diversity was observed among the groups, with results ranging from 0.71 to 2.73. These findings correlate with predictions from the Simpson index, with values ranging from 0.10 to 0.83 (with 2 outlier values above 0.75), indicating a high degree of microbial community diversity and instability in the hBM microbial community. No statistically significant differences were observed between groups (Fig 3).

### Beta diversity analysis

**Fig 4:**
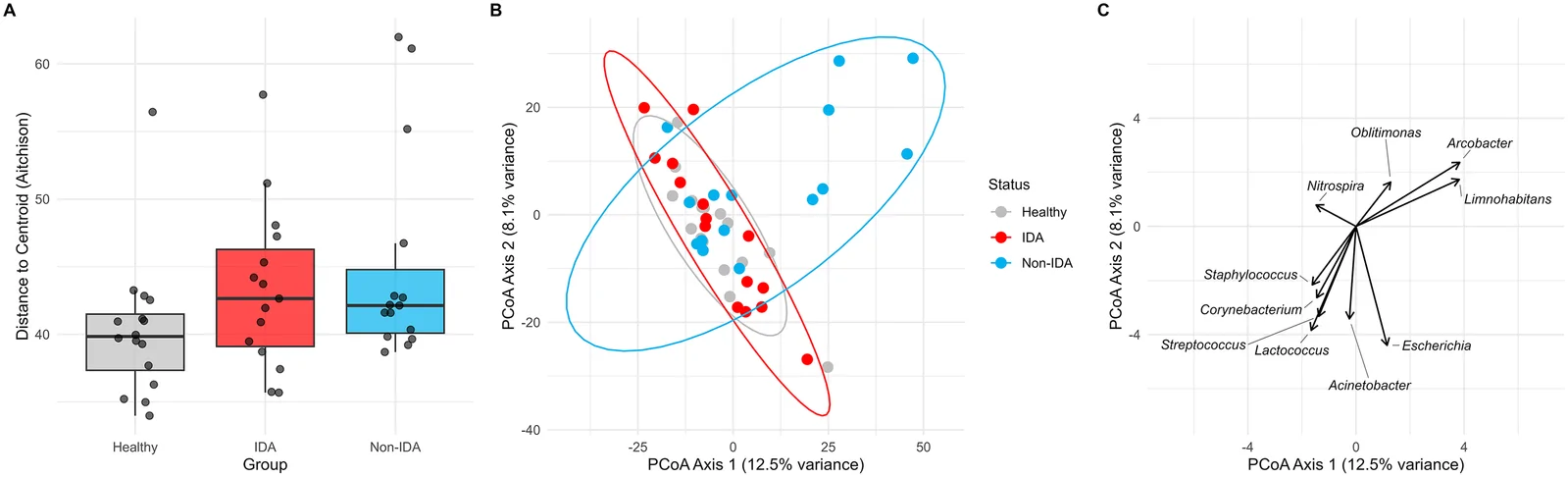
Principal Coordinates Analysis Biplot and Selected Genera Vectors.

Beta diversity was assessed among healthy, IDA and non-IDA participants. Principal coordinate analysis using Aitchison distance showed similar distribution patterns for healthy and IDA groups, with substantial overlap between samples. In contrast, the non-IDA group exhibited greater dispersion along the PCoA1 axis. Statistically significant differences were detected using PERMANOVA (p = 0.008), with anaemia status explaining 6.3% of the variance (R² = 0.063), indicating a modest but measurable effect size (Fig 4).

### Differences according to anaemia status and 12 most common bacterial genera

**Fig 5:**
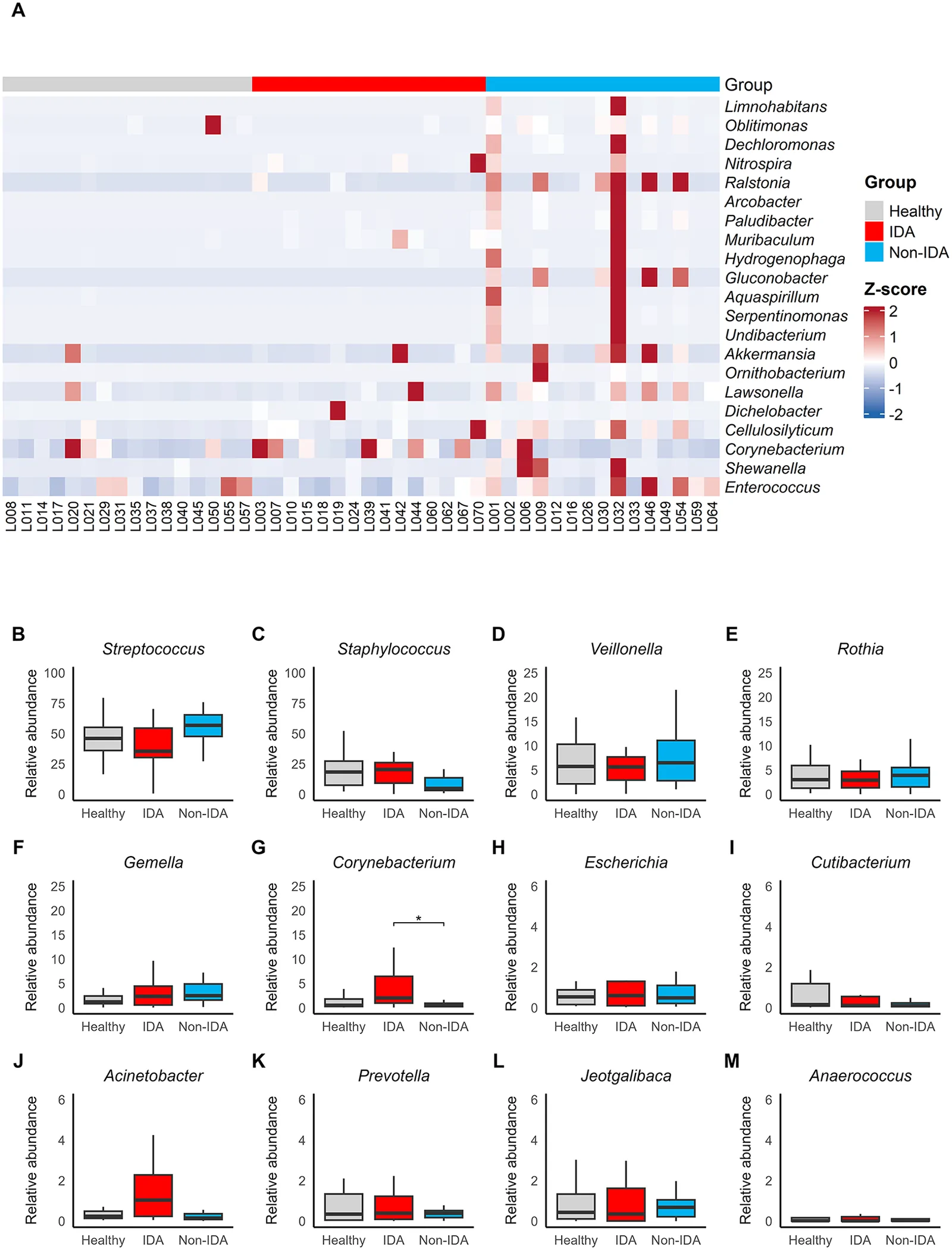
Heatmap representation of frequencies of selected bacterial genera.

*Streptococcus spp.* was the predominant bacterial genus across most samples, representing 45.0% (range: 6.51–79.47%), 39.6% (range: 0.70–70.37%) and 55.9% (range: 27.25–75.94%) in the healthy, IDA and non-IDA groups, respectively. A trend toward lower abundance was observed in the IDA group; however, no statistically significant differences were detected between groups. *Staphylococcus spp.* was the second most abundant genus, with an overall mean relative abundance of 19.57% (range: 0.26–80.26%) across all samples (Fig 5)

Out of the 12 most common bacterial genera across all samples, only *Corynebacterium*, an aerobic, gram-positive bacillus, yielded a statistically significant difference between the IDA and non-IDA group. IDA participants were observed to have a mean frequency of 3.76% in hBM samples, with values ranging from 0.06 to 12.40%. These values were higher than those from healthy group (mean frequency = 1.68%) and IDA group, which showed a mean frequency of 2.01% (p-value < 0.05) (Fig 5).

Additionally, statistically significant differences were observed between healthy and IDA groups for frequencies of *Muribaculum* (p-value < 0.01), *Nitrospira* (p-value < 0.01) and *Paludibacter* (p-value < 0.05) (Fig 5).

Overall, relatively few bacterial genera differentiated IDA participants from the healthy group. In contrast, the non-IDA group exhibited a broader pattern of taxonomic variation. Compared with healthy participants, 15 bacterial genera showed statistically significant differences in relative abundance (Supplementary Data, S5 Table), including *Akkermansia*, *Limnohabitans*, *Paludibacter* and *Ralstonia*, which displayed the strongest statistical associations (p < 0.01). These findings suggest greater microbial heterogeneity within the non-IDA group, although the biological significance of these differences remains uncertain.

Further analysis did not reveal any bacterial genera significantly correlated with haemoglobin concentration. However, some bacterial populations showed weak positive associations with maternal age and gestational age at birth (Supplementary Data).

## Discussion

The primary objective of this study was to investigate potential associations between hBM microbiota and iron deficiency anaemia (IDA). To better contextualize findings potentially related to iron deficiency, we included both healthy participants and children with non-IDA as comparison groups.

A recent official report by the Peruvian Ministry of Health showed that over 33% of children below 3 years of age had anaemia [6]. Since iron deficiency is the most common cause of anaemia worldwide [3], identifying factors associated with iron deficiency anaemia remains a major public health priority. Although multiple studies have focused on the socio-economic determinants of anaemia [5], less attention has been given to biological factors that may contribute to its development. In this context, the human breast milk microbiota has emerged as a potential factor associated with infant anaemia and iron homeostasis.

The microbial composition of hBM has shown some variability across populations, depending on geographical, genetic, environmental, and maternal health-related characteristics [9]. For example, Ward et al. studied the microbiome in hBM samples from 10 women in Canada using 16S rRNA analysis, finding a dominance of *Pseudomonas spp,* which represented 61% of abundance [25]*.* On the other hand, a pilot study conducted in California (USA) on a cohort of Mexican-American woman identified the genus *Streptococcus* as the most abundant, representing 73.8% of the total bacterial composition [26]. Davé et al. highlighted differences with other studies [26], like Cabrera-Rubio et al., which reported that *Lactobacillus* was the most abundant genus within a Finnish population [27]. Nevertheless, a recent review by Dombrowska-Pali et al., discussed the finding of 16 articles on hBM microbiome and reported that *Streptococcus*, *Staphylococcus*, *Serratia*, *Pseudomonas*, *Corynebacterium*, *Ralstonia*, *Cutibacterium*, *Sphingomonas* and *Bradyrhizobium* are consistently identified as the predominant genera contributing to hBM microbial composition [9].

In our study, we detected 213 genera across 46 hBM samples with *Streptococcus* and *Staphylococcus* being the most abundant, contributing to 46.8% and 19.6% of the bacterial population, respectively. *Veillonella*, *Rothia*, *Corynebacterium* and *Gemella* also contributed significantly, which shows consistency with the core hBM microbiome reported in the literature [9]. These findings align with results from the INSPIRE study, which reported that breast milk samples from Peruvian mothers displayed a mean abundance of approximately 50% *Streptococcus*, significantly higher than in other cohorts (p ≤ 0.0386) [28]. In comparative analyses, Peru clustered with Spain, characterized by high abundance of *Streptococcus, Staphylococcus*, and *Corynebacterium* [28]. This geographical clustering suggests that regional and cultural factors may exert a strong influence on hBM microbiota composition. In this context, the predominance of *Streptococcus* and *Staphylococcus* in our cohort is consistent with patterns observed in Peruvian populations, reinforcing the value of interpreting microbiome data within a geographically contextualized framework.

In relation to the microbial population of anaemic participants, the PCoA plot (Fig 4) revealed no clear separation between healthy and IDA participants, with substantial overlap in microbial community distribution. While the Non-IDA group showed a wider dispersion and partial divergence from the other groups, this pattern was not consistent enough to indicate strong differentiation. PCoA1 and PCoA2 explained only 12.5% and 8.1% of the variance, respectively, indicating that the anaemia-related grouping accounted for just a minor proportion of the overall microbiota variability. Although statistically significant, the modest effect size (R² = 0.063) indicates that anaemia status contributes to microbiota variability but is not a dominant determinant of overall community structure, supporting a multifactorial model of hBM microbiota composition.

However, the inclusion of both healthy and non-IDA comparison groups provided a useful framework for distinguishing microbiota patterns potentially associated with iron deficiency from those associated with anaemia more broadly. Differential abundance analysis revealed specific taxonomic differences between groups, suggesting that subtle microbial shifts may occur even in the absence of marked community-level separation.

Alpha diversity was assessed with Chao1, Shannon and Simpson indexes. Analysis of Chao1 index revealed a statistically significant higher value in patients with non-IDA versus Healthy participants (p < 0.01) and the IDA group (p < 0.05). Because the Chao1 index primarily reflects species richness and is particularly sensitive to low-abundance taxa, these findings suggest that non-IDA participants may contain a greater number of low-abundance bacterial taxa. Nevertheless, the persistence of dominant genera, like *Streptococcus* and *Staphylococcus*, across all the participants in the study, may reflect the lack of statistically significant differences in the Shannon and Simpson indexes (Fig 3). These findings are also compatible with PCoA analysis, which showed a wider spread across PC1 for non-IDA participants, without clustering differentiation (Fig 4).

Furthermore, when analysing taxonomic abundance, the IDA group displayed a statistically higher relative abundance of three genera, *Muribaculum* (p = 0.005), *Nitrospira* (p = 0.003), and *Paludibacter* (p = 0.02), when compared with healthy individuals. To further explore potential relationships between microbial abundance and iron status, correlations between ferritin levels and bacterial genera were evaluated. Significant positive correlations were observed between ferritin concentration and the relative abundance of *Oblitimonas*, *Limnohabitans*, *Dechloromonas* and *Enterococcus* (Supplementary Data, S7 Fig).

In comparison with non-IDA group, IDA participants showed significantly higher frequency of *Corynebacterium* (p-value = 0.04) and lower frequency for several genera, including *Enterococcus* and *Gluconobacter* (see Supplementary Data). However, the relative abundances were low in most cases, except for *Corynebacterium* (Fig 5A and Supplementary data).

*Corynebacterium* has been commonly reported in hBM, particularly during early stages of lactation, suggesting a potential role in infant gut colonization [29]. Importantly, *Corynebacterium* was the only highly abundant genus showing significant differences between IDA and non-IDA participants. In contrast, a trend towards a lower mean relative abundance of *Streptococcus* was observed in the IDA group (39.64%), compared with healthy participants (45.05%) and those in the non-IDA group (55.86%). Although this difference did not reach statistical significance, the lower relative abundance observed in IDA participants was considered noteworthy given the predominance of *Streptococcus* within the hBM microbiota. *Streptococcus spp*. is a highly abundant and recurrent bacterial genus within the hBM microbiota across different populations worldwide [30]. Its presence is largely attributed to the retrograde flow of the infant’s oral microbiota during suckling [31]. Furthermore, *Streptococcus spp*. are dominant colonizers of the oral cavity and, to a lesser extent, may be introduced through sebum-type skin flora located around the nipple area [32]. Interestingly, although iron deficiency has been associated with an increased abundance of *Streptococcus* spp. in the oral environment [33], we observed a lower relative abundance of this genus in hBM from mothers of IDA children. Given that *Streptococcus* spp. in breast milk are primarily derived from the infant’s oral microbiota through retrograde transfer during suckling, this finding likely reflects differences in microbial transfer dynamics between the oral cavity and the mammary gland, rather than a direct effect of iron availability on hBM composition.

Notably, this trend appeared to follow an inverse pattern to that observed for *Corynebacterium*. Although the correlation between both genera did not reach statistical significance (Spearman R = –0.41, p = 0.15), previous studies have reported clinically relevant interactions between them [34]. Specifically, *Corynebacterium* has been shown to inhibit *Streptococcus* growth in both *in vitro* and *in vivo* respiratory models [34], and increased *Corynebacterium* abundance in the respiratory tract has been associated with lower *Streptococcus pneumoniae* burden and reduced infection risk [35–37]. These observations, together with our findings, suggest that interactions between *Corynebacterium* and *Streptococcus* may contribute to the microbial patterns observed in IDA participants. Nevertheless, due to the limited sample size and absence of mechanistic analysis, further studies with larger and longitudinal cohorts are required to confirm this association and elucidate its biological relevance.

*Lactobacillus* is a genus that has been frequently reported in hBM and its potential role in the development of a healthy gut microbiota has been highlighted in multiple studies [38]. Likewise, lactic acid bacteria *Bifidobacterium sp*., have been shown to be associated to beneficial gut microbiome [10]. However, in our study, none of these genera were detected in meaningful proportions. *Lactobacillus* represented 0.17%, ranging between 1.16 to 0.04% across all samples and without statistically significant differences between groups. For *Bifidobacterium*, the mean relative abundance across all samples was 0.12% (range: 0–2.24%), showing no statistically significant differences between groups. hBM is known to adapt its composition to meet the nutritional needs of the infant [39]. Although *Bifidobacterium* has been associated with improved gut health and enhanced nutrient absorption [12], our analysis did not reveal any differential pattern for this or related genera between children with IDA.

Interestingly, we detected *Jeotgalibaca* spp., which represented 0.8% of microbial abundance (range: 0–3.03%), ranking among the 12 most abundant genera identified in our cohort. To our knowledge, *Jeotgalibaca* has not previously been reported in human breast milk. Although no meaningful associations with demographic or microbial variables were identified in the present study, this finding may warrant further investigation. Previous studies have reported *Jeotgalibaca* as a component of the milk microbiota of farm animals [40,41], while more recent reports have identified this genus in the human gut microbiota [42]. Additionally, associations between *Jeotgalibaca* abundance and host metabolic or inflammatory markers have been described in specific clinical settings [42]. Given the recognized role of inflammatory pathways in iron homeostasis [43], these observations highlight the limited but emerging evidence regarding this genus. However, its biological significance in the human breast milk microbiome remains unknown and should be interpreted cautiously until confirmed in larger studies.

The non-IDA group exhibited statistically significant differences in 15 bacterial genera when compared with healthy participants, whereas only three genera differed between IDA and healthy participants (Fig 5 and Supplementary Data). Because non-IDA represents a heterogeneous category encompassing multiple conditions with distinct pathophysiological mechanisms, the biological interpretation of these findings remains uncertain. Nevertheless, these observations suggest that microbiota variation associated with anaemia may differ according to the underlying aetiology and support the importance of distinguishing iron deficiency anaemia from other forms of anaemia in microbiome studies.

It should also be noted that although milk samples were collected using standard aseptic procedures, no parallel sampling or characterization of the areolar skin microbiota was performed. Therefore, we cannot completely exclude a potential contribution from maternal skin flora to the microbial profile detected in hBM, particularly for genera such as *Staphylococcus* and *Cutibacterium*. Future studies incorporating paired skin microbiota sampling would enhance interpretation of microbial origin.

Another limitation relates to the absence of adjustment for potential confounding factors such as maternal diet, breastfeeding modality (exclusive vs. mixed feeding), or lifestyle variables, which may influence microbial composition. Furthermore, while statistically significant differences were identified at the taxonomic level, the relatively small effect sizes observed in beta diversity analyses indicate subtle microbial variations, which should be interpreted with caution.

Finally, the sequencing approach used did not enable species-level resolution, a relevant constraint considering the high abundance of *Streptococcus* detected in hBM. This genus includes both potentially beneficial species—such as *Streptococcus salivarius* and *Streptococcus parasanguinis*, associated with anti-inflammatory effects and gut colonisation [44]—as well as opportunistic pathogens such as *Streptococcus agalactiae*, linked to severe neonatal disease [45]. Future research incorporating longitudinal sampling, species-level metagenomic profiling, and parallel infant microbiome analysis would be essential to determine whether specific *Streptococcus* lineages contribute to protective or pathogenic effects in this context.

## Conclusions

This study explored the potential association between IDA in children and the microbial composition of hBM. Although beta diversity analyses indicated that anaemia status explained only a small proportion of the overall microbiota variability, taxonomic analyses identified specific differences associated with IDA, including increased relative abundance of *Corynebacterium* and a trend toward lower abundance of *Streptococcus.* These findings suggest that iron deficiency anaemia may be associated with subtle alterations in hBM microbiota composition, despite the overall conservation of community structure.

While the present study was not designed to establish causal relationships, the observed associations support further investigation of hBM microbiota as a potential biological factor associated with iron deficiency anaemia in early childhood. Future studies incorporating larger cohorts, longitudinal designs and functional microbiome analyses will be necessary to determine the biological significance of these associations and their potential contribution to iron homeostasis and anaemia development.

## Supporting information

S1 Table Socio-demographics of enrolled participants according to anaemia status. (DOCX)

S2 Table Laboratory results according to clinical status. (DOCX)

S3 Fig Top 30 most common bacterial genera across all samples. (TIFF)

S4 Fig Rarefaction curves for Healthy (top), IDA (middle) and non-IDA (bottom) samples. (TIFF)

S5 Table Bacterial genera with statistically significant differences between study groups. (DOCX)

S6 Fig Positive correlations of bacterial genera. (TIFF)

S7 Fig Statistically significant correlations of Corynebacterium with other bacterial genera. (TIFF)

S8 Fig Correlations of ferritin levels with bacterial genera. (TIFF)
