# Supplementary material for: Breastmilk microbiota and its association with infant iron deficiency anaemia: A 16S rRNA metagenomic study in Peruvian mothers cohort
Source: PLoS One. 2026 Aug 27;21(8):e0352428. doi: 10.1371/journal.pone.0352428 (PMC13521397; doi:10.1371/journal.pone.0352428)
Supplement: Supplementary file 1 [file pone.0352428.s009.pdf]

General comments: Thank you for the opportunity to review this paper. Overall, I recommend the authors review the manuscript for consistency in italicizing bacterial species and family names. Additionally, the discussion section is limited and should be expanded to clearly explain the relevance of the bacterial taxa identified in the IDA and non-IDA groups. This will help strengthen the impact and significance of the study.

Title: "Peruvian mothers" is broad, and implies national-level representation. I suggest you consider …" *Peruvian mother cohort*"

Abstract: This should be 16S rRNA sequencing.

Line 46-54: Although this briefly mentions anemia rates in Peru, I recommend placing greater emphasis on national and regional anemia statistics, particularly in Piura, the city where the participants were recruited.

Line 50-55: The authors mention that socioeconomic inequalities contribute to anemia but then transition to a discussion on the gut microbiota without clearly explaining the connection. I suggest they explicitly state that differences in socioeconomic status can lead to poor dietary intake, which may in turn affect gut microbiota composition and subsequently influence breast milk microbiota. Additionally, more emphasis should be placed on breast milk microbiota rather than gut microbiota, given the focus of the study.

Line 54: Please indicate which regions in Peru.

Line 55-56, 65-66: The authors state that the gut microbiota plays a crucial role and mention that iron levels can alter the intestinal microbiota in adult women. However, the connection between iron status and gut microbiota is not clearly explained. Also, this paper focuses on breast milk microbiota, not gut microbiota. However, the authors include information about the gut microbiota without clearly linking it to the study's main focus. I recommend placing greater emphasis on breast milk microbiota and its influence on the infant's gut microbiota, while limiting discussion on the gut microbiota itself unless it is directly relevant and clearly integrated into the study's context.

Line 68-71: This section needs to be more specific. The significance of the microbial groups described here is not explained. What roles do these bacteria play in infant health or development? I recommend the authors elaborate on the functional relevance of these taxa to provide more meaningful context.

Line 72: Please clarify, is it "healthy" or "apparent healthy" women.

Line 81: The manuscript should provide more specific information regarding the health status of the mothers, how was this assessed or defined? Additionally, since anemia is more prevalent in populations with limited access to resources and malnutrition, it is important to clarify whether these factors were considered. Breast milk composition can be influenced by various

environmental and lifestyle factors, please specify which factors were controlled for or accounted for in the study.

Line 99-100: Which types of growth and cognitive measures were collected? The current description is too general.

Line 103: Were fecal samples collected from infants? If so, this should be clearly stated here. Also, how long were the samples stored at room temperature? Additionally, it should be clarified whether fecal samples were collected solely for parasite identification. If that is the case, please state this clearly in the methods section.

Line 106: I suggest the authors explain why this method was chosen. Was this method selected for reasons of convenience, accessibility, or another justification? Please clarify in the methods section.

Line 110: Provide references on the method used.

Line 113: More details are needed regarding breast milk collection. Was the sample collected at a specific time of day or stage of feeding? Were participants instructed to maintain their regular diets and routines prior to sample collection? Please clarify these aspects to better understand the consistency and context of the samples.

Line 177-179: Why were these factors chosen for analysis? Please clarify. What about other environmental factors such as maternal diet, socioeconomic status, or lifestyle? These variables are likely more relevant and could have a greater impact on both anemia and breast milk composition. I recommend the authors consider including or discussing these factors.

Line 180: anemia is a repeated word

Line 201: need to correct to “per”

Line 203-204: Based on the WHO reference cited in the paper, this study included children with mild to moderate anemia, please clarify.

Line 210: The term “Blastocystis hominis” needs to be italicized. Additionally, it should be clearly indicated that this organism is a parasite and explain why it was evaluated in the study. Even if this seems obvious to the authors, providing these details will help readers understand its relevance.

Line 211: not clear what (n) means for children with IDA and non-IDA.

Line 215: Change determine to determined

Line 229: Use past tense: “*was* observed” instead of “*is* observed”

Line 267: Could you please clarify what you are comparing the significant differences in healthy participants to? The current statement is unclear.

Line 271-274: Please revise the grammar here to make this statement clearer and easier to understand. I suggest: "*Further analysis did not identify any bacterial genus correlated with hemoglobin concentration. However, some bacterial populations showed weak positive correlations with demographic characteristics, such as maternal age and gestational age at birth."* Check if this is what you want to express.

Line 287: *Streptococcus* should be italicized. In general, I recommend the authors review the entire manuscript to ensure that bacterial genera and families are consistently and correctly formatted.

Line 288: Is "Dave" a reference? If so, it needs to be properly indicated.

Line 301-303: please clarify which groups you are referring to.

Line 315: Please specify what IL-1β refers to. While this may be obvious to the authors, I suggest clarifying that it is a pro-inflammatory cytokine. Also, I will please explain the relevance of including this statement in relation to your findings.

Line 326-327: please indicate the role or significance of *Muribaculum*, *Nitrospira*, and *Paludibacter* in breast milk or infant health.

Line 331: This statement is quite general. Please elaborate on which specific pathologies are included under "multiple pathologies" in the non-IDA group.

Line 333-334: Please revise the sentence to accurately reflect the observed differences and help readers understand the reduction you are referring to.

Line 348-355. Even though the authors have clearly stated the limitations of this study, I encourage them to discuss the relevance of their results especially for the population of Piura. For instance, how might these findings inform early detection strategies, nutritional interventions, or maternal health programs in this region? Your study provides an important evidence base that contributes to the scientific understanding of breast milk microbiota in relation to anemia, and has the potential to support and guide future community-based interventions, health policy planning, and targeted research efforts in low-resource settings.

Line 354: change "specie" to "species"

Line 355: The statement regarding the *Streptococcus* genus is too general. Please clarify the relevance of this genus in the context of your study, why is its presence or abundance important?

Line 360: Same as comment above, please clarify the relevance of *Corynebacterium* in IDA.

Conclusion section is missing.
