# Supplementary material for: Breastmilk microbiota and its association with infant iron deficiency anaemia: A 16S rRNA metagenomic study in Peruvian mothers cohort
Source: PLoS One. 2026 Aug 27;21(8):e0352428. doi: 10.1371/journal.pone.0352428 (PMC13521397; doi:10.1371/journal.pone.0352428)
Supplement: Supplementary file 2 [file pone.0352428.s001.docx]

**S1 Table. Sociodemographic characteristics of enrolled participants according to anaemia status**

|  | **Healthy n=16 (%)** | **IDA n=15 (%)** | **Non-IDA n=15 (%)** | **p-value (a)** |
| --- | --- | --- | --- | --- |
| **Type of housing** |  |  |  |  |
| House | 50 | 46.7 | 86.7 | 0.064 |
| Apartment | 0 | 0 | 6.7 |  |
| Hut | 37.5 | 33.3 | 0 |  |
| Improvised shelter | 12.5 | 20 | 6.7 |  |
| **Floor material** |  |  |  |  |
| Wood | 6.3 | 6.7 | 6.7 | 0.857 |
| Synthetic/vynil flooring | 6.3 | 6.7 | 0 |  |
| Tiles | 6.3 | 13.3 | 26.7 |  |
| Cement | 62.5 | 66.7 | 60 |  |
| Dirt | 12.5 | 6.7 | 6.7 |  |
| **Numbers of rooms** | 2.6 +/- 1.2 | 2.2 +/- 1.1 | 2.6 +/- 1.7 | 0.668 (b) |
| **Numbers of bedrooms** | 2.44 +/- 1.4 | 2.3 +/- 1.1 | 2.1 +/- 1.2 | 0.798 (c) |
| **Housing status** |  |  |  |  |
| Rented | 0 | 20 | 20 | 0.481 |
| Owned, legally | 37.5 | 26.7 | 46.7 |  |
| Informal settlement | 31.3 | 26.7 | 13.3 |  |
| Other | 31.3 | 26.7 | 20 |  |
| **Water supply** | **Healthy n=16 (%)** | **IDA n=15 (%)** | **Non-IDA n=15 (%)** | **p-value (a)** |
| Public water network | 75 | 60 | 80 | 0.292 |
| Public water pump | 18.8 | 6.7 | 13.3 |  |
| Tank truck | 12.5 | 20 | 6.7 |  |
| River, lake, etc. | 6.3 | 0 | 0 |  |
| Other | 0 | 13.3 | 0 |  |
| **Sewer** |  |  |  |  |
| Public standpipe | 68.8 | 60 | 73.3 | 0.923 |
| Septic tank | 6.3 | 6.7 | 6.7 |  |
| River, lake, etc. | 12.5 | 13.3 | 6.7 |  |
| Open field | 6.3 | 13.3 | 0 |  |
| Other | 6.3 | 6.7 | 13.3 |  |
| **Fuel for cooking** |  |  |  |  |
| Liquefied petroleum gas | 93.8 | 86.7 | 93.3 | 0.739 |
| Charcoal | 6.3 | 13.3 | 6.7 |  |
| **Telecommunication** |  |  |  |  |
| Telephone | 100 | 100 | 93.3 | 0.348 |
| Cell phone | 6.2 | 6.7 | 6.7 | 0.99 |
| Cable TV | 46.7 | 33.3 | 26.7 | 0.602 |
| Internet | 86.7 | 86.7 | 66.7 | 0.388 |

“Categorical variables are presented as n (%) and continuous variables as mean ± standard deviation or median (interquartile range), as appropriate. Comparisons were performed using (a) Pearson’s chi-square test or Fisher’s exact test, (b) one-way ANOVA, and (c) the Kruskal–Wallis test.”
