# Supplementary material for: Breastmilk microbiota and its association with infant iron deficiency anaemia: A 16S rRNA metagenomic study in Peruvian mothers cohort
Source: PLoS One. 2026 Aug 27;21(8):e0352428. doi: 10.1371/journal.pone.0352428 (PMC13521397; doi:10.1371/journal.pone.0352428)
Supplement: Supplementary file 3 [file pone.0352428.s002.docx]

**S2 Table. Laboratory results according to clinical status.**

|  | **Healthy** | **IDA** | **Non-IDA^b^** |
| --- | --- | --- | --- |
| Infant haemoglobin (g/dL) |  |  |  |
| 3-5 months (n=10) | 10.9 +/- 0.8 | - | 9.4 +/- 0.0 |
| 6-12 months (n=36) | 12.3 +/- 0.9 | 9.9 +/- 0.6 | 9.8 +/- 0.9 |
| Ferritin (µg/L)^a^ | - | 3.4 +/- 3.9 | 61.1 +/- 28.9 |
| Maternal haemoglobin (g/dL) (g/dL) (n=46) | 12.6 +/- 1.1 | 13.1 +/- 1.1 | 12.5 +/- 0.8 |
| *Blastocystis hominis* (% positive) | 0 | 6.7 | 12.5 |

*Values are presented as mean ± standard deviation unless otherwise indicated, —: no participants. ^a^Ferritin was measured only in children who met the diagnostic criteria for anaemia. ^b^Non-IDA was defined as anaemia without biochemical evidence of iron deficiency based on ferritin concentration
