# Supplementary material for: Breastmilk microbiota and its association with infant iron deficiency anaemia: A 16S rRNA metagenomic study in Peruvian mothers cohort
Source: PLoS One. 2026 Aug 27;21(8):e0352428. doi: 10.1371/journal.pone.0352428 (PMC13521397; doi:10.1371/journal.pone.0352428)
Supplement: Supplementary file 4 [file pone.0352428.s005.docx]

**S5 Table. Bacterial genera with statistically significant differences between study groups.**

|  | **Genus** | **Group1** | **Group2** | **Global Kruskal -Wallis p** | **Adjusted**  **pairwise p** |
| --- | --- | --- | --- | --- | --- |
| 1 | *Akkermansia* | Non-IDA | Healthy | 0.014 | 0.010 |
| 2 | *Aquaspirillum* | Non-IDA | Healthy | 0.011 | 0.038 |
| 3 | *Arcobacter* | Non-IDA | Healthy | 0.004 | 0.019 |
| 4 | *Cellulosilyticum* | Non-IDA | Healthy | 0.029 | 0.026 |
| 5 | *Corynebacterium* | Non-IDA | IDA | 0.033 | 0.044 |
| 6 | *Dechloromonas* | Non-IDA | Healthy | 0.001 | 0.013 |
| 7 | *Dechloromonas* | Non-IDA | IDA | 0.001 | 0.017 |
| 8 | *Dichelobacter* | Non-IDA | Healthy | 0.028 | 0.033 |
| 9 | *Enterococcus* | Non-IDA | IDA | 0.039 | 0.034 |
| 10 | *Gluconobacter* | Non-IDA | IDA | 0.009 | 0.037 |
| 11 | *Hydrogenophaga* | Non-IDA | Healthy | 0.007 | 0.025 |
| 12 | *Lawsonella* | Non-IDA | Healthy | 0.014 | 0.018 |
| 13 | *Limnohabitans* | Non-IDA | Healthy | 0.000 | 0.005 |
| 14 | *Limnohabitans* | Non-IDA | IDA | 0.000 | 0.007 |
| 15 | *Muribaculum* | IDA | Healthy | 0.007 | 0.005 |
| 16 | *Nitrospira* | IDA | Healthy | 0.001 | 0.003 |
| 17 | *Nitrospira* | Non-IDA | IDA | 0.001 | 0.016 |
| 18 | *Oblitimonas* | Non-IDA | Healthy | 0.001 | 0.021 |
| 19 | *Oblitimonas* | Non-IDA | IDA | 0.001 | 0.003 |
| 20 | *Ornithobacterium* | Non-IDA | IDA | 0.014 | 0.032 |
| 21 | *Paludibacter* | IDA | Healthy | 0.007 | 0.019 |
| 22 | *Paludibacter* | Non-IDA | Healthy | 0.007 | 0.008 |
| 23 | *Ralstonia* | Non-IDA | Healthy | 0.001 | 0.002 |
| 24 | *Serpentinomonas* | Non-IDA | Healthy | 0.013 | 0.044 |
| 25 | *Shewanella* | Non-IDA | Healthy | 0.035 | 0.049 |
| 26 | *Undibacterium* | Non-IDA | Healthy | 0.013 | 0.044 |

*Overall differences among the three groups were assessed using the Kruskal–Wallis test. Pairwise comparisons were performed using Dunn’s test, and p values were adjusted using the Bonferroni method.”
