# Supplementary material for: Breastmilk microbiota and its association with infant iron deficiency anaemia: A 16S rRNA metagenomic study in Peruvian mothers cohort
Source: PLoS One. 2026 Aug 27;21(8):e0352428. doi: 10.1371/journal.pone.0352428 (PMC13521397; doi:10.1371/journal.pone.0352428)
Supplement: Supplementary file 7 [file pone.0352428.s013.docx]

PONE-D-25-28555R2

Breastmilk Microbiota and Its Association with Infant Anaemia: A 16S rRNA Metagenomic Study in Peruvian Mothers

PLOS One

Dear Dr. Gonzales,

Thank you for submitting your manuscript to PLOS ONE. After careful consideration, we feel that it has merit but does not fully meet PLOS ONE’s publication criteria as it currently stands. Therefore, we invite you to submit a revised version of the manuscript that addresses the points raised during the review process.

==============================

**ACADEMIC EDITOR:** The manuscript addresses an important topic and shows considerable effort. However, clearer structuring of findings across the healthy, IDA, and non-IDA groups, improved figure readability, and a more concise interpretation of microbiota changes are needed to strengthen the manuscript.

==============================

Please submit your revised manuscript by Jul 08 2026 11:59PM. If you will need more time than this to complete your revisions, please reply to this message or contact the journal office at [plosone@plos.org](mailto:plosone@plos.org). When you're ready to submit your revision, log on to https://www.editorialmanager.com/pone/ and select the 'Submissions Needing Revision' folder to locate your manuscript file.

Please include the following items when submitting your revised manuscript:

- A letter that responds to each point raised by the academic editor and reviewer(s). You should upload this letter as a separate file labeled 'Response to Reviewers'.
- A marked-up copy of your manuscript that highlights changes made to the original version. You should upload this as a separate file labeled 'Revised Manuscript with Track Changes'.
- An unmarked version of your revised paper without tracked changes. You should upload this as a separate file labeled 'Manuscript'.

If you would like to make changes to your financial disclosure, please include your updated statement in your cover letter. Guidelines for resubmitting your figure files are available below the reviewer comments at the end of this letter.

If applicable, we recommend that you deposit your laboratory protocols in protocols.io to enhance the reproducibility of your results. Protocols.io assigns your protocol its own identifier (DOI) so that it can be cited independently in the future. For instructions see: <https://journals.plos.org/plosone/s/submission-guidelines#loc-laboratory-protocols>. Additionally, PLOS ONE offers an option for publishing peer-reviewed Lab Protocol articles, which describe protocols hosted on protocols.io. Read more information on sharing protocols at <https://plos.org/protocols?utm_medium=editorial-email&utm_source=authorletters&utm_campaign=protocols>.

As the corresponding author, your ORCID iD is verified in the submission system and will appear in the published article. PLOS supports the use of ORCID, and we encourage all coauthors to register for an ORCID iD and use it as well. Please encourage your coauthors to verify their ORCID iD within the submission system before final acceptance, as unverified ORCID iDs will not appear in the published article. *Only* the individual author can complete the verification step; PLOS staff *cannot* verify ORCID iDs on behalf of authors.

We look forward to receiving your revised manuscript.

Kind regards,

Bijit Biswas, MBBS, MD, DNB
Academic Editor
PLOS One

**Journal Requirements:**

1. If the reviewer comments include a recommendation to cite specific previously published works, please review and evaluate these publications to determine whether they are relevant and should be cited. There is no requirement to cite these works unless the editor has indicated otherwise. 

2. Please review your reference list to ensure that it is complete and correct. If you have cited papers that have been retracted, please include the rationale for doing so in the manuscript text, or remove these references and replace them with relevant current references. Any changes to the reference list should be mentioned in the rebuttal letter that accompanies your revised manuscript. If you need to cite a retracted article, indicate the article’s retracted status in the References list and also include a citation and full reference for the retraction notice.

**Additional Editor Comments:**

The manuscript addresses an important topic and shows considerable effort. However, clearer structuring of findings across the healthy, IDA, and non-IDA groups, improved figure readability, and a more concise interpretation of microbiota changes are needed to strengthen the manuscript.

***We appreciate the editor’s suggestions and have revised the manuscript accordingly.***

1. *Clearer structuring of findings*

***The Results and Discussion sections were reorganized to more clearly differentiate findings among healthy, IDA and non-IDA groups. Additional text was added to explain the role of the comparison groups and to maintain the focus on the primary objective of investigating associations between hBM microbiota and IDA.***

2. ***Improved figure readability***

***All figures were regenerated at higher resolution and exported using publication-quality settings. Font sizes, labels and legends were enlarged to improve readability both on screen and in print.***

3. ***More concise interpretation of microbiota changes***

***Several discussion sections were shortened and revised to reduce speculative interpretation. Conclusions were also rewritten to better reflect the exploratory nature of the study, emphasizing associations rather than causal relationships.***

***We believe these modifications have substantially strengthened the manuscript and improved the clarity and interpretation of the findings.***

***All figures were regenerated and reformatted to improve readability. Font sizes, labels, legends and graphical elements were enlarged, and higher-resolution figure files were prepared to ensure adequate visualization both on screen and in printed format. Figure legends were also revised to improve clarity and facilitate interpretation.***

[Note: HTML markup is below. Please do not edit.]

Reviewers' comments:

Reviewer's Responses to Questions

**Comments to the Author**

1. If the authors have adequately addressed your comments raised in a previous round of review and you feel that this manuscript is now acceptable for publication, you may indicate that here to bypass the “Comments to the Author” section, enter your conflict of interest statement in the “Confidential to Editor” section, and submit your "Accept" recommendation.

Reviewer #2: All comments have been addressed

Reviewer #4: (No Response)

2. Is the manuscript technically sound, and do the data support the conclusions?

The manuscript must describe a technically sound piece of scientific research with data that supports the conclusions. Experiments must have been conducted rigorously, with appropriate controls, replication, and sample sizes. The conclusions must be drawn appropriately based on the data presented.

Reviewer #2: Yes

Reviewer #4: Partly

3. Has the statistical analysis been performed appropriately and rigorously?

Reviewer #2: Yes

Reviewer #4: Yes

4. Have the authors made all data underlying the findings in their manuscript fully available?

The [PLOS Data policy](http://www.plosone.org/static/policies.action#sharing) requires authors to make all data underlying the findings described in their manuscript fully available without restriction, with rare exception (please refer to the Data Availability Statement in the manuscript PDF file). The data should be provided as part of the manuscript or its supporting information, or deposited to a public repository. For example, in addition to summary statistics, the data points behind means, medians and variance measures should be available. If there are restrictions on publicly sharing data—e.g. participant privacy or use of data from a third party—those must be specified.

Reviewer #2: Yes

Reviewer #4: Yes

5. Is the manuscript presented in an intelligible fashion and written in standard English?

PLOS ONE does not copyedit accepted manuscripts, so the language in submitted articles must be clear, correct, and unambiguous. Any typographical or grammatical errors should be corrected at revision, so please note any specific errors here.

Reviewer #2: Yes

Reviewer #4: Yes

6. Review Comments to the Author

Please use the space provided to explain your answers to the questions above. You may also include additional comments for the author, including concerns about dual publication, research ethics, or publication ethics. (Please upload your review as an attachment if it exceeds 20,000 characters)

Reviewer #2: The authors have responded well to the previous comments, and the manuscript is now clearer and more balanced in its interpretation. The exploratory nature of the study is appropriately emphasized, and the presentation of the results has improved. Overall, I have no further major concerns and believe the manuscript is suitable for publication.

Reviewer #4: I acknowledge that the authors have invested a huge amount of work in this project.
However, I still have a few remarks.
1) Lines 165-170, Bioformatics analysis: The authors use an abbreviation not explained -- "OTU". I suggest explaining the abbreviation.

***Corrected***
2) The title is appropriate that breastmilk microbiota is associated with infant anaemia. But this is where the follwing text becomes confusing for me. The authors have defined 3 groups: healthy, IDA and non-IDA. The authors conclude that IDA showed significant increased corynebacteria and reduced streptococci frequencies, i.e. they are stressing the difference in regard to iron deficiency. However, they repeatedly report throughout the text that bacterial changes were noted to a lesser extent between IDA and healthy participants but more between the IDA plus healthy groups versus non-IDA. It may help if the authors were to structure their results and discussion according to the 3 groups they formed. At the moment, the text is very specialised discussing each bacterium.

***We thank the reviewer for this important observation. We agree that the previous version of the manuscript did not sufficiently explain the rationale for including the non-IDA group and may have inadvertently shifted attention away from the primary objective of the study.***

***To address this concern, we have substantially revised the Introduction, Methods, Results and Discussion sections.***

***First, we explicitly clarified that the primary objective of the study was to investigate potential associations between human breast milk (hBM) microbiota and iron deficiency anaemia (IDA), while the non-IDA and healthy groups were included as comparison groups to distinguish microbiota patterns potentially associated with iron deficiency from those associated with anaemia more broadly.***

***Accordingly:***

- ***The Introduction now explains the rationale for including healthy and non-IDA comparison groups.***
- ***The Methods section explicitly states that the non-IDA and healthy groups were incorporated to facilitate identification of microbiota characteristics potentially associated with iron deficiency rather than anaemia status alone.***
- ***The Results section was reorganized to clearly distinguish findings observed between IDA and healthy participants, IDA and non-IDA participants, and healthy and non-IDA participants.***
- ***The Discussion was substantially restructured to emphasize the primary IDA-related findings while still acknowledging the broader taxonomic variation observed in the non-IDA group.***
- ***Additional text was incorporated to explain that non-IDA represents a heterogeneous category and that interpretation of those findings should therefore be made with caution.***

***Furthermore, we reduced speculative interpretations and focused the discussion on the principal findings directly related to IDA, namely the increased abundance of Corynebacterium, the lower abundance trend observed for Streptococcus, and the overall modest but measurable microbiota differences associated with iron deficiency anaemia. We believe these revisions have substantially improved the clarity of the manuscript and better align the interpretation of the findings with the original study objective.***

3) I cannot read the figures and legends and the print out is also unclear. They are too small and too blurred.

***All figures were regenerated and reformatted to improve readability. Font sizes, labels, legends and graphical elements were enlarged, and higher-resolution figure files were prepared to ensure adequate visualization both on screen and in printed format. Figure legends were also revised to improve clarity and facilitate interpretation.***

7. PLOS authors have the option to publish the peer review history of their article ([what does this mean?](https://journals.plos.org/plosone/s/editorial-and-peer-review-process#loc-peer-review-history)). If published, this will include your full peer review and any attached files.

If you choose “no”, your identity will remain anonymous but your review may still be made public.

**Do you want your identity to be public for this peer review?** For information about this choice, including consent withdrawal, please see our [Privacy Policy](https://www.plos.org/privacy-policy).

Reviewer #2: No

Reviewer #4: No

[NOTE: If reviewer comments were submitted as an attachment file, they will be attached to this email and accessible via the submission site. Please log into your account, locate the manuscript record, and check for the action link "View Attachments". If this link does not appear, there are no attachment files.]

To ensure your figures meet our technical requirements, please review our figure guidelines: https://journals.plos.org/plosone/s/figures

You may also use PLOS’s free figure tool, NAAS, to help you prepare publication quality figures: https://journals.plos.org/plosone/s/figures#loc-tools-for-figure-preparation.

NAAS will assess whether your figures meet our technical requirements by comparing each figure against our figure specifications.
