# Supplementary material for: Breastmilk microbiota and its association with infant iron deficiency anaemia: A 16S rRNA metagenomic study in Peruvian mothers cohort
Source: PLoS One. 2026 Aug 27;21(8):e0352428. doi: 10.1371/journal.pone.0352428 (PMC13521397; doi:10.1371/journal.pone.0352428)
Supplement: Supplementary file 8 [file pone.0352428.s011.docx]

*5. Review Comments to the Author

Please use the space provided to explain your answers to the questions above. You may also include additional comments for the author, including concerns about dual publication, research ethics, or publication ethics. (Please upload your review as an attachment if it exceeds 20,000 characters)*

*Reviewer #1: General comments: Thank you for the opportunity to review this paper. Overall, I recommend the authors review the manuscript for consistency in italicizing bacterial species and family names. Additionally, the discussion section is limited and should be expanded to clearly explain the relevance of the bacterial taxa identified in the IDA and non-IDA groups. This will help strengthen the impact and significance of the study.
Title: “Peruvian mothers” is broad, and implies national-level representation. I suggest you consider …” Peruvian mother cohort”*

***Corrected*** *Abstract: This should be 16S rRNA sequencing.*

***Corrected*** *Line 46-54: Although this briefly mentions anemia rates in Peru, I recommend placing greater emphasis on national and regional anemia statistics, particularly in Piura, the city where the participants were recruited.*

***Information about Piura was included.*** *Line 50-55: The authors mention that socioeconomic inequalities contribute to anemia but then transition to a discussion on the gut microbiota without clearly explaining the connection. I suggest they explicitly state that differences in socioeconomic status can lead to poor dietary intake, which may in turn affect gut microbiota composition and subsequently influence breast milk microbiota. Additionally, more emphasis should be placed on breast milk microbiota rather than gut microbiota, given the focus of the study.*

***Corrected*** *Line 54: Please indicate which regions in Peru.*

***Corrected: The case of Piura was specified*** *Line 55-56, 65-66: The authors state that the gut microbiota plays a crucial role and mention that iron levels can alter the intestinal microbiota in adult women. However, the connection between iron status and gut microbiota is not clearly explained. Also, this paper focuses on breast milk microbiota, not gut microbiota. However, the authors include information about the gut microbiota without clearly linking it to the study's main focus. I recommend placing greater emphasis on breast milk microbiota and its influence on the infant’s gut microbiota, while limiting discussion on the gut microbiota itself unless it is directly relevant and clearly integrated into the study's context.*

***Corrected: The paragraphs have been revised to more clearly introduce the role of the human breastmilk microbiome*** *Line 68-71: This section needs to be more specific. The significance of the microbial groups described here is not explained. What roles do these bacteria play in infant health or development? I recommend the authors elaborate on the functional relevance of these taxa to provide more meaningful context.*

***Corrected: The paragraphs have been revised*** *Line 72: Please clarify, is it “healthy” or “apparent healthy” women.*

***Corrected: It was specified*** *Line 81: The manuscript should provide more specific information regarding the health status of the mothers, how was this assessed or defined? Additionally, since anemia is more prevalent in populations with limited access to resources and malnutrition, it is important to clarify whether these factors were considered. Breast milk composition can be influenced by various environmental and lifestyle factors, please specify which factors were controlled for or accounted for in the study.*

***According to the manuscript, maternal health status—which determined eligibility for inclusion in the study—was defined based on the following criteria: recent antibiotic use, concurrent chemotherapy, HIV infection, and the presence of mammary gland inflammation. Additionally, socioeconomic homogeneity between study groups was ensured using a modified questionnaire that collected information on housing conditions, energy source, water source, and home ownership status (owned or rented), all of which were comparable across groups, without significant intergroup differences. This information is described in detail in the manuscript.***

*Line 99-100: Which types of growth and cognitive measures were collected? The current description is too general.*

***Corrected: The text was clarified, indicating that only information on birth weight and length was collected. This data was obtained from the “Healthy Child Checkup Card,” an official registry of the Ministry of Health.*** *Line 103: Were fecal samples collected from infants? If so, this should be clearly stated here. Also, how long were the samples stored at room temperature? Additionally, it should be clarified whether fecal samples were collected solely for parasite identification. If that is the case, please state this clearly in the methods section.*

***Corrected: It was specified that stool samples were collected exclusively from the infants for parasitological analysis, and that they were stored at room temperature.***

*Line 106: I suggest the authors explain why this method was chosen. Was this method selected for reasons of convenience, accessibility, or another justification? Please clarify in the methods section.*

***Corrected: The use of a portable hemoglobinometer was selected primarily for practical and logistical reasons, as haemoglobin*** ***measurements*** ***were conducted directly in the field during community-based visits. This approach ensured immediate assessment of anaemia status in infants, without the need for laboratory infrastructure. We have clarified this rationale in the Methods section.***

*Line 110: Provide references on the method used.*

***Corrected*** *Line 113: More details are needed regarding breast milk collection. Was the sample collected at a specific time of day or stage of feeding? Were participants instructed to maintain their regular diets and routines prior to sample collection? Please clarify these aspects to better understand the consistency and context of the samples.*

***Corrected: It was specified***

*Line 177-179: Why were these factors chosen for analysis? Please clarify. What about other environmental factors such as maternal diet, socioeconomic status, or lifestyle? These variables are likely more relevant and could have a greater impact on both anemia and breast milk composition. I recommend the authors consider including or discussing these factors.*

***Socioeconomic status was assessed in all participants using a modified version of the National Demographic and Family Health Survey (ENDES), and the nutritional status of the infants was also evaluated for the purpose of determining whether these factors could influence anaemia status across study groups. No statistically significant differences were observed between groups for any of these variables. Although the inclusion of maternal dietary assessment would have provided additional insight—particularly in relation to breast milk composition—it was not evaluated in this study. The methodological decision was based on the fact that, in our setting, dietary assessment tools are subject to high variability and may not accurately reflect micronutrient intake, whereas haemoglobin levels provide a more direct and objective indicator of maternal iron status. For this reason, maternal haemoglobin concentration was used as the primary proxy for nutritional status related to anaemia risk. We have incorporated this clarification into the revised manuscript.***

*Line 180: anemia is a repeated word*

***Corrected*** *Line 201: need to correct to “per”*

***Corrected*** *Line 203-204: Based on the WHO reference cited in the paper, this study included children with mild to moderate anemia, please clarify.*

***Corrected*** *Line 210: The term “Blastocystis hominis” needs to be italicized. Additionally, it should be clearly indicated that this organism is a parasite and explain why it was evaluated in the study. Even if this seems obvious to the authors, providing these details will help readers understand its relevance.*

***Corrected and expanded***

*Line 211: not clear what (n) means for children with IDA and non-IDA.*

***Corrected*** *Line 215: Change determine to determined*

***Corrected*** *Line 229: Use past tense: “was observed” instead of “is observed”*

***Corrected*** *Line 267: Could you please clarify what you are comparing the significant differences in healthy participants to? The current statement is unclear.*

***Corrected*** *Line 271-274: Please revise the grammar here to make this statement clearer and easier to understand. I suggest: “Further analysis did not identify any bacterial genus correlated with hemoglobin concentration. However, some bacterial populations showed weak positive correlations with demographic characteristics, such as maternal age and gestational age at birth.” Check if this is what you want to express.*

***Corrected***

*Line 287: Streptococcus should be italicized. In general, I recommend the authors review the entire manuscript to ensure that bacterial genera and families are consistently and correctly formatted.*

***Corrected***

*Line 288: Is “Dave” a reference? If so, it needs to be properly indicated.*

*Line 301-303: please clarify which groups you are referring to.*

***Corrected***

*Line 315: Please specify what IL-1β refers to. While this may be obvious to the authors, I suggest clarifying that it is a pro-inflammatory cytokine. Also, I will please explain the relevance of including this statement in relation to your findings.*

***Corrected: The discussion has been expanded***

*Line 326-327: please indicate the role or significance of Muribaculum, Nitrospira, and Paludibacter in breast milk or infant health.*

***Corrected: The discussion has been expanded***

*Line 331: This statement is quite general. Please elaborate on which specific pathologies are included under “multiple pathologies” in the non-IDA group.*

***Corrected: The discussion has been expanded***

*Line 333-334: Please revise the sentence to accurately reflect the observed differences and help readers understand the reduction you are referring to.*

***Corrected: The discussion has been expanded***

*Line 348-355. Even though the authors have clearly stated the limitations of this study, I encourage them to discuss the relevance of their results especially for the population of Piura. For instance, how might these findings inform early detection strategies, nutritional interventions, or maternal health programs in this region? Your study provides an important evidence base that contributes to the scientific understanding of breast milk microbiota in relation to anemia, and has the potential to support and guide future community-based interventions, health policy planning, and targeted research efforts in low-resource settings.*

***Corrected: The discussion has been expanded***

*Line 354: change “specie” to “species”*

***Corrected***

*Line 355: The statement regarding the Streptococcus genus is too general. Please clarify the relevance of this genus in the context of your study, why is its presence or abundance important?*

***Corrected: The discussion has been expanded***

*Line 360: Same as comment above, please clarify the relevance of Corynebacterium in IDA.*

***Corrected: The discussion has been expanded***

*Conclusion section is missing.*

***Corrected***

*Reviewer #2: This study investigates the association between breast milk microbiota and anaemia subtypes in infants (IDA and non-IDA), which is a novel and relevant research question with evident public health importance. The use of 16S rRNA sequencing is a strength, and the focus on a vulnerable population makes the work meaningful. The findings on increased microbial richness in non-IDA and the enrichment of Corynebacterium in IDA are interesting and could provide the basis for future hypotheses.
However, the manuscript requires major revision to reach publication quality. The sample size is small (n=46, divided into three groups), and multiple comparisons are reported without clear statistical adjustment, raising concerns about reliability. Applying correction for multiple testing and reporting effect sizes would strengthen confidence in the results. The Results section is overly descriptive; the text should focus on the main findings, with detailed genus-level data moved to supplementary material. Figures require simplification, correction of typographical errors, and a more explicit focus on biologically relevant taxa. The Discussion is well-referenced but sometimes speculative. For example, the suggestion of compensatory microbiota strategies should be presented as a hypothesis rather than a conclusion. The limitations section is helpful but could be expanded to acknowledge the single time point design, the absence of infant gut microbiota data, potential confounders such as maternal diet or breastfeeding mode, and the relatively small effect sizes seen in beta diversity analysis. In addition, the manuscript requires careful language editing to improve clarity and flow, and the novelty of the work should be emphasised more strongly in the Introduction. Finally, there are inconsistencies in font size across the manuscript (some in 11 pt and others in 12 pt), which should be standardised.*

- ***Regarding the statistical considerations, we fully acknowledge the concern raised about the absence of correction for multiple comparisons. However, given the exploratory and pilot nature of our study, the limited statistical power due to the sample size, and the hypothesis-generating objectives, we intentionally opted not to apply strict correction methods such as FDR or Bonferroni. Applying such adjustments in this context could lead to an increased risk of type II errors, masking biologically relevant signals that may be valuable for guiding future research. This decision aligns with current recommendations in microbiome statistics, which emphasise prioritising trend-based interpretation over rigid significance thresholds in exploratory datasets (Gloor et al., Front Microbiol, 2017). Similarly, Noyan et al. (Nat Rev Methods Primers, 2022) highlight that in pilot studies of microbiome research, overly conservative statistical corrections can be counterproductive when the primary aim is hypothesis generation rather than confirmatory inference.***
- ***We appreciate the reviewer observation; however, given the exploratory nature of the study and the limited availability of comparable research in this population, we consider it essential to retain genus-level descriptive detail in the Results section. This information is necessary to adequately contextualise the findings and support hypothesis generation, which is a core objective of the study. Therefore, we have preserved this level of detail to maintain scientific interpretability and transparency.***
- ***The discussion has been revised and expanded to enhance its depth and overall contribution***
- ***The limitations section has been revised to explicitly acknowledge the single time-point design, the absence of infant gut microbiota data, the potential influence of confounding factors such as maternal diet and breastfeeding mode, and the relatively small effect sizes observed in beta diversity analysis. These points have now been clearly integrated, reinforcing the exploratory nature of the study and supporting cautious interpretation of the results.***
- ***We have revised and strengthened the introduction, including improvements to formatting and narrative flow, as well as refining the clarity of the discussion sections.***

*Reviewer #3: There is great potential in the research work, but it lacks several basic information that needs to be addressed.
1. Based on manual stimulation of skin, did you consider normal flora of skin around the breasts that can be introduced as part of the sample being collected and how was that issue addressed in your methodology?*

***Thank you for this important observation. We agree that skin-associated microbiota may contribute to the microbial profile detected in breast milk samples, particularly in genera such as Staphylococcus* and *Cutibacterium. In our study, milk samples were collected following standard aseptic procedures consistent with protocols commonly used in microbiome research; however, we did not perform specific sampling of the skin surface (e.g., swabbing of the areolar region) for comparison. Consequently, we were unable to differentiate the extent of contribution from cutaneous microbiota versus endogenous milk microbiota.***

***We acknowledge that this constitutes a methodological limitation, which has now been explicitly incorporated into the* Limitations *section. Future studies would benefit from parallel sampling of the maternal skin microbiota to improve interpretation of microbial origin, particularly for taxa commonly associated with the skin.***

*2. Using the hemoglobinometer for Hemoglobin estimation, what was the safeguard protocol implemented to*

*ensure accurate Hemoglobin estimation if veinous blood sample was collected and assessed using a compact full blood count analyzer.*

***No automated full blood count analysis was performed. Hemoglobin concentration was assessed exclusively using a portable hemoglobinometer, as this measurement was used as an inclusion screening criterion. Due to the very young age of the participants and considering the ethical and practical constraints of obtaining venous blood from all children during field recruitment, capillary sampling with hemoglobinometer testing was deemed the most appropriate approach. To ensure the accuracy of the measurements, a manufacturer-provided control solution was applied at the beginning of each field sampling session to validate device performance.***

*3. Did you ensure babies were not on any antibiotics before taking stool samples to avoid interference with accurate results.*

**Antibiotic exposure was not controlled in infants. The only exclusion criteria related to antibiotic use were applied to the mothers. Since stool samples were not collected from the children in this study, and the microbiota analysis focused exclusively on human breast milk (hBM), no direct evaluation of infant gut microbiota was performed. Therefore, restricting antibiotic exposure in infants was not required for the microbiome outcomes investigated.**

*4. Was the breast milk stored immediately after collection, kept at room temperature for a specified period of time before refrigeration, etc since these factors can affect the microbiota of the breast milk?*

**Breast milk samples were refrigerated immediately after collection at 4 °C and maintained under this condition for a maximum of 2 hours. Subsequently, they were stored at −20 °C for no longer than 48 hours before being transferred to −80 °C for long-term preservation. These procedures were implemented to minimise potential alterations in microbial composition due to temperature variation and are now clearly specified in the revised manuscript.**

*5. How was the purity of the extracted DNA samples determined, any immunoblot testing done?
These issues need to be addressed to reflect the scope of work done as extensive and scientifically viable since your sample size is the bare minimum and does not really have a good representation of the health concerns being raised to affect populations in Peru.*

**The purity and concentration of the extracted DNA were evaluated using spectrophotometric quantification , which provides high specificity for double-stranded DNA. Spectrophotometric ratios (A260/280) were monitored and remained within acceptable ranges for metagenomic sequencing (1.8–2.0). Immunoblot analysis was not performed, as our study focused on genotypic profiling through 16S rRNA sequencing and not on protein expression mechanisms. Protein-level assessment would have required additional experimental procedures (e.g., induction assays and specific antibody-based detection), beyond the scope and primary objectives of this work.**

*Reviewer #4: To the authors:
Page 1, Abstract, line14 and Page 2, Introduction, line 41: „is estimated 1.8 billion….“. Please define the number: people? Dollars?*
***Corrected***

*Page 2, Abstract, lines 35-36: „… with IDA and non-IDA…“ The whole article revolves around the difference between healthy and IDA infants on the one side and non-IDA infants on the other. Thus, it may be more descriptive to rephrase the title. Furthermore, this would also suggest reviewing the keywords (line 38)as they concentrate on IDA infants.*

***We appreciate the reviewer’s suggestion. However, the current title and keywords were intentionally retained, as the focus on non-IDA accurately reflects the analytical approach of the study. Non-IDA was emphasized due to its broader and less predictable pathophysiology compared to IDA, which allowed a more specific and detailed exploration of microbiota associations. This strategy resulted in the identification of a higher number of significant genera in the non-IDA group, supporting the relevance of maintaining the current wording***

*Page 3, Introduction, line 3: „Children that have suffered anaemia…“ I suggest „Children that suffer from anaemia …*
***Corrected***

*Page 3, Introduction, line 55: „In that respect, the gut microbiota plays a crucial role…“ I suggest „In that respect, the gut microbiota may play a crucial role…“ The whole article builds up tot he conclusion that iron deficiency anaemia is not related microbiota, whereas non-iron deficiency is relatated. The arising question: What is behind „non-iron deficiency anaemia“ ?*

***We thank the reviewer for the suggestion. The introduction was revised following prior reviewer feedback to strengthen the role of breastmilk microbiota. While IDA is primarily related to iron metabolism, non-IDA may result from other nutritional deficiencies (e.g., vitamin B12, folate), inflammatory processes or haematological conditions. Although our findings do not allow definitive conclusions regarding microbiota involvement in IDA, the differential abundance of genera such as* Corynebacterium *and* Streptococcus *suggests a potential role that warrants further investigation under study designs not subject to our current limitations.***

*Page 3, Introduction, line 60: „ … acids (SCFAs), which contributes to the …“ I suggest „„ … acids (SCFAs), which contribute to the …“*
***Corrected: The introduction was revised following prior reviewer feedback to strengthen the role of breastmilk microbiota.***

*Page 6, Collection of breastmilk, lines 113-114: „After the confirmation that the participant…“ I suggest „After confirmation that the participant…“*
***Corrected: has been modified and expanded*** *Page 9, Ethics, lines 164-168. I suggest moving the paragraph up in the text to page 6 before Laboratory.*
***Corrected*** *Page 10, Demographics, line 182: „ …18 and 39 years, with an mea nage of 27,6.“ I suggest „18 to 30 years, with an mean age of 27.6“*

***Corrected*** *Page 10, Demographics, line 184: „...birth was a significally …“ I suggest „… birth was significally…“*

***Corrected*** *Page 10, Demographics, line 188: The authors refer to „differences observed between the groups“. However, they have not explained to which groups they are refering. The definition follows later on page 11.*
***Corrected*** *Page 11, Demographics, lines 203 to 209: The authors do not state the number of childrenin each group.*
***Corrected*** *Page 12, Microbial richness in Hbm, line 225: „… population, Mean values for healthy participants was …“ I suggest „… population, Mean values for healthy participants were …“*
***Corrected*** *Page 12, Microbial richness in Hbm, line 229: „… High degree of diversity is observed…“ I suggest „… High degree of diversity was observed…“*
***Corrected*** *Page 12, Differences … bacterial genera“, lines 253– 254: A verb is missing in the last sentence. I suggest rephrasing the whole paragraph.*
***Corrected*** *Pages 14 – 18, Discussion: The text presently focusses on the bacterial findings without any connection to possible effects of the bacterial colonisation. The authors start their submission with an explanation why iron defiency is important (lines 55-64). But instead of a clinical translation of their findings, they resort to a basic mathematical listing of bacteria without consequences.*

***We appreciate the reviewer comment. The Discussion was deliberately structured to present the microbial findings before introducing potential clinical implications, in line with the exploratory nature of the study. Although cautious in interpretation, we did highlight relevant biological hypotheses—for example, the potential compensatory role of Corynebacterium in IDA and the reduced abundance of Streptococcus, possibly linked to alterations in breastfeeding frequency or oral microbiota transfer (pages 15–16). These exploratory associations were intentionally presented without overstatement due to study limitations such as sample size and lack of mechanistic validation. Rather than a purely mathematical listing, our aim was to provide microbiological evidence that may guide future studies assessing clinical impact more directly***

*Page 16, Discussion, line 307: „… we also detected a Jeotgalibaca sp…“ I suggest „… we also detected Jeotgalibaca sp…“*
***Corrected*** *In general: What is being subsumated as non-iron deficiency. And where do the authors' readout tie in with the clinical situations and consequences. At present, they are disputing any involvement in microbiota and iron deficiency-- which contradicts the build-up of the introduction.*

*6. PLOS authors have the option to publish the peer review history of their article (*[*what does this mean?*](https://journals.plos.org/plosone/s/editorial-and-peer-review-process#loc-peer-review-history)*). If published, this will include your full peer review and any attached files.

If you choose “no”, your identity will remain anonymous but your review may still be made public.*

***Do you want your identity to be public for this peer review?*** *For information about this choice, including consent withdrawal, please see our* [*Privacy Policy*](https://www.plos.org/privacy-policy)*.*

*Reviewer #1: No*

*Reviewer #2: No*

*Reviewer #3:* ***Yes:*** *PRINCE ANIM ADDO*

*Reviewer #4:* ***Yes:*** *Dr. Patrick Biggar*
