# Supplementary material for: Breastmilk microbiota and its association with infant iron deficiency anaemia: A 16S rRNA metagenomic study in Peruvian mothers cohort
Source: PLoS One. 2026 Aug 27;21(8):e0352428. doi: 10.1371/journal.pone.0352428 (PMC13521397; doi:10.1371/journal.pone.0352428)
Supplement: Supplementary file 9 [file pone.0352428.s012.docx]

| **Date:** | Mar 04 2026 11:41PM |
| --- | --- |
| **To:** | "Arturo Gonzales" arturo.gonzales@udep.edu.pe |
| **From:** | "PLOS ONE" plosone@plos.org |
| **Subject:** | PLOS ONE Decision: Revision required [PONE-D-25-28555R1] |

PONE-D-25-28555R1

Breastmilk Microbiota and Its Association with Infant Anaemia: A 16S rRNA Metagenomic Study in Peruvian Mothers

PLOS One

Dear Dr. Gonzales,

Thank you for submitting your manuscript to PLOS ONE. After careful consideration, we feel that it has merit but does not fully meet PLOS ONE’s publication criteria as it currently stands. Therefore, we invite you to submit a revised version of the manuscript that addresses the points raised during the review process.

==============================
**ACADEMIC EDITOR:** The manuscript has improved following revision and addresses an interesting research question. However, minor revisions are still needed. The conclusions should remain cautious and reflect the exploratory nature and small sample size of the study. The Results and Discussion sections could be streamlined to focus on the most relevant findings, and minor language and clarity issues should be corrected.
==============================

Please submit your revised manuscript by Apr 18 2026 11:59PM. If you will need more time than this to complete your revisions, please reply to this message or contact the journal office at [plosone@plos.org](mailto:plosone@plos.org). When you're ready to submit your revision, log on to https://www.editorialmanager.com/pone/ and select the 'Submissions Needing Revision' folder to locate your manuscript file.

Please include the following items when submitting your revised manuscript:

- A letter that responds to each point raised by the academic editor and reviewer(s). You should upload this letter as a separate file labeled 'Response to Reviewers'.
- A marked-up copy of your manuscript that highlights changes made to the original version. You should upload this as a separate file labeled 'Revised Manuscript with Track Changes'.
- An unmarked version of your revised paper without tracked changes. You should upload this as a separate file labeled 'Manuscript'.

If you would like to make changes to your financial disclosure, please include your updated statement in your cover letter. Guidelines for resubmitting your figure files are available below the reviewer comments at the end of this letter.

If applicable, we recommend that you deposit your laboratory protocols in protocols.io to enhance the reproducibility of your results. Protocols.io assigns your protocol its own identifier (DOI) so that it can be cited independently in the future. For instructions see: <https://journals.plos.org/plosone/s/submission-guidelines#loc-laboratory-protocols>. Additionally, PLOS ONE offers an option for publishing peer-reviewed Lab Protocol articles, which describe protocols hosted on protocols.io. Read more information on sharing protocols at <https://plos.org/protocols?utm_medium=editorial-email&utm_source=authorletters&utm_campaign=protocols>.

We look forward to receiving your revised manuscript.

Kind regards,

Bijit Biswas, MBBS, MD, DNB
Academic Editor
PLOS One

Journal Requirements:

If the reviewer comments include a recommendation to cite specific previously published works, please review and evaluate these publications to determine whether they are relevant and should be cited. There is no requirement to cite these works unless the editor has indicated otherwise. 

Please review your reference list to ensure that it is complete and correct. If you have cited papers that have been retracted, please include the rationale for doing so in the manuscript text, or remove these references and replace them with relevant current references. Any changes to the reference list should be mentioned in the rebuttal letter that accompanies your revised manuscript. If you need to cite a retracted article, indicate the article’s retracted status in the References list and also include a citation and full reference for the retraction notice.

Additional Editor Comments:

The manuscript has improved following revision and addresses an interesting research question. However, minor revisions are still needed. The conclusions should remain cautious and reflect the exploratory nature and small sample size of the study. The Results and Discussion sections could be streamlined to focus on the most relevant findings, and minor language and clarity issues should be corrected.


[Note: HTML markup is below. Please do not edit.]

Reviewers' comments:

Reviewer's Responses to Questions

**Comments to the Author**

1. If the authors have adequately addressed your comments raised in a previous round of review and you feel that this manuscript is now acceptable for publication, you may indicate that here to bypass the “Comments to the Author” section, enter your conflict of interest statement in the “Confidential to Editor” section, and submit your "Accept" recommendation.

Reviewer #1: All comments have been addressed

Reviewer #2: (No Response)

Reviewer #3: All comments have been addressed

Reviewer #4: (No Response)

2. Is the manuscript technically sound, and do the data support the conclusions?

The manuscript must describe a technically sound piece of scientific research with data that supports the conclusions. Experiments must have been conducted rigorously, with appropriate controls, replication, and sample sizes. The conclusions must be drawn appropriately based on the data presented.

Reviewer #1: Yes

Reviewer #2: Partly

Reviewer #3: Yes

Reviewer #4: No

3. Has the statistical analysis been performed appropriately and rigorously?

Reviewer #1: Yes

Reviewer #2: Yes

Reviewer #3: Yes

Reviewer #4: Yes

4. Have the authors made all data underlying the findings in their manuscript fully available?

The [PLOS Data policy](http://www.plosone.org/static/policies.action#sharing) requires authors to make all data underlying the findings described in their manuscript fully available without restriction, with rare exception (please refer to the Data Availability Statement in the manuscript PDF file). The data should be provided as part of the manuscript or its supporting information, or deposited to a public repository. For example, in addition to summary statistics, the data points behind means, medians and variance measures should be available. If there are restrictions on publicly sharing data—e.g. participant privacy or use of data from a third party—those must be specified.

Reviewer #1: Yes

Reviewer #2: Yes

Reviewer #3: Yes

Reviewer #4: Yes

5. Is the manuscript presented in an intelligible fashion and written in standard English?

PLOS ONE does not copyedit accepted manuscripts, so the language in submitted articles must be clear, correct, and unambiguous. Any typographical or grammatical errors should be corrected at revision, so please note any specific errors here.

Reviewer #1: Yes

Reviewer #2: Yes

Reviewer #3: Yes

Reviewer #4: Yes

6. Review Comments to the Author

Please use the space provided to explain your answers to the questions above. You may also include additional comments for the author, including concerns about dual publication, research ethics, or publication ethics. (Please upload your review as an attachment if it exceeds 20,000 characters)

Reviewer #1: The authors have implemented relevant revisions that have improved the manuscript. However, the conclusion section should be clearly presented, unless it is considered an optional component under the journal’s formatting guidelines.

***We thank the reviewer for this helpful suggestion. Although PLOS ONE does not require a separate conclusion section, we agree that a clearly defined closing statement improves the clarity of the manuscript. We have therefore added a dedicated “Conclusions” section summarizing the main findings and their implications.***

Reviewer #2: The authors have addressed the main points raised in the previous round of review, and the manuscript has improved in clarity and focus. The exploratory and pilot nature of the study is now more clearly articulated, and the limitations section has been expanded to acknowledge key constraints, including the small sample size, the single time-point design, and potential confounding factors.
The study is technically sound and addresses a novel and relevant research question regarding the association between breast milk microbiota and anaemia subtypes in infants. The conclusions are generally supported by the data, provided that the findings are consistently interpreted within an exploratory, hypothesis-generating framework.
A small number of minor points should still be addressed before acceptance. Any remaining language suggesting causality or definitive conclusions should be revised to better reflect the exploratory design. Reporting effect sizes, where feasible, would strengthen interpretation by allowing assessment of biological relevance independent of statistical significance. In addition, the Results section could be further streamlined by emphasizing the most biologically relevant taxa, with more detailed genus-level descriptions moved to the Supplementary Material. Figures and formatting should also be carefully reviewed to ensure clarity and consistency.
Overall, only minor revisions are required, and addressing these points will further improve the clarity and scientific presentation of the manuscript.

***We thank the reviewer for these constructive and insightful comments. First, we have carefully revised the manuscript to ensure that all language suggesting causality has been replaced with terminology more consistent with the exploratory nature of the study. Expressions implying definitive conclusions have been modified to reflect association-based interpretations.bSecond, we have strengthened the interpretation of our findings by incorporating effect size considerations where appropriate, including measures of variance explained in beta diversity analyses and clearer reporting of relative abundance differences. Third, the Results and Discussion section has been streamlined to emphasize the most biologically relevant taxa. Detailed descriptions of additional genera have been moved to the Supplementary Material to improve clarity and readability. Finally, all figures and formatting have been carefully reviewed and revised to ensure consistency, clarity, and alignment with journal standards.***

Reviewer #3: THIS IS A GOOD SCIENTIFIC RESEARCH PROJECT WITH GREAT PERSPECTIVES FOR FURTHER STUDIES ON SIMILAR RESEARCH

Reviewer #4: Abstract, line 19: “46 mother-child pairs were recruited from Talara, a town in the northern coast of Peru…“ I suggest “ 46 mother-child pairs were recruited from Talara, a town on the northern coast of Peru …“

***Corrected***
Introduction, line 46: “For several decades, the initiatives to reduce…“ I suggest “ For several decades, initiatives to reduce…“
***Corrected***
Introduction, line 68: “ contributes to the degradation and uptake of human milk oligosaccharides (HMOs), through…“ I suggest deleting the comma „contributes to the degradation and uptake of human milk oligosaccharides (HMOs) through….“
***Corrected***
Introduction, lines 81/82: “ In this study, we analysed the breastmilk microbiota of apparent healthy women within …“ I suggest “ In this study, we analysed the breastmilk microbiota of apparently healthy women within…“
***Corrected***
Population, line 89: “ … of Peru, recruitment was conducted from October 4 to December 20, 2019.“ I suggest replacing the comma with either a semicolon or starting a new sentence, e.g. “ … of Peru. Recruitment was conducted from October 4 to December 20, 2019.“
***Corrected***
Population, lines 89/90: “ The inclusion criteria included children between 3 …“ Repetition of inclusion. I suggest “Inclusion criteria comprised children between 3…“ and deleting “The..“
***Corrected***
Demographics, line 228: “… mean Ferritin concentration of 3.4 μg/L.“ I suggest “ mean ferritin concentration of 3.4 μg/L.“
***Corrected***
Microbial richness in hBM, line 249: “… population, Mean values…“ I suggest “… population, mean values…“
***Corrected***
Differences according to anaemia status and 12 most common bacterial genera, line 282/283: “… only Corynebacterium, an aerobic, gram-positive bacilli, yielded …“ Either sigular or plural, but not both. I suggest “ only Corynebacterium, an aerobic, gram-positive bacillus, yielded …“

***Corrected***

Discussion, lines 320/321: “ In our study, we detected 213 genera across 46 hBM samples, being Streptococcus and Staphylococcus the most abundant, contributing to…“ I suggest „In our study, we detected 213 genera across 46 hBM samples with Streptococcus and Staphylococcus being the most abundant, contributing to…“
***Corrected***

Discussion, lines 375-379: “ Interestingly, during iron deficiency, reduced bioavailability of iron in the oral environment has been associated with increased frequency of Streptococcus spp. [33]. In this context, we hypothesise that the reduced abundance of Streptococcus spp. in hBM from mothers of IDA children may reflect diminished breastfeeding frequency and/or duration, …“ These sentences are contradictory: first, iron deficiency has been associated with increased Strept. spp. Then second, reduced strept. spp. may reflect IDA. Please explain.

Furthermore, “we hypothesise“ should read “we hypothesised“.

Also, the statement “… may reflect diminished breastfeeding frequency and/or duration,“ has the potential of destroying this complete paper.

***We thank the reviewer for this insightful comment. We agree that, as originally phrased, the sentences may appear contradictory and require clarification.***

***First, the observation that iron deficiency has been associated with an increased abundance of Streptococcus spp. refers specifically to the oral environment, where reduced iron availability may favour the growth of certain bacterial taxa. In contrast, our findings relate to the human breast milk (hBM) microbiota, which represents a distinct ecological niche influenced by multiple factors beyond systemic or local iron status.***

***Importantly, the presence of Streptococcus spp. in hBM has been consistently attributed to the retrograde transfer of the infant’s oral microbiota during suckling, rather than direct modulation by iron availability within the mammary gland. Therefore, the lower relative abundance observed in hBM from mothers of IDA children does not contradict previous findings, but rather suggests that the efficiency of microbial transfer between the infant oral cavity and breast milk may be altered.***

***To address the reviewer’s concern, we have revised the text to clarify that our hypothesis does not attribute the observed difference directly to iron deficiency per se, but to potential modulation of microbial transfer dynamics.***

***Regarding the statement on breastfeeding frequency and duration, we agree that this requires careful wording. Our intention was not to introduce an unsupported causal claim, but to propose a biologically plausible explanation consistent with the known mechanism of retrograde flow. We have therefore softened this statement to reflect its exploratory nature and to avoid overinterpretation.***

***Finally, we have corrected “we hypothesise” to “we hypothesised” as suggested.***

Discussion, total and especially line 262: “… Although no clear separation in overall microbial structure was observed between study groups, differences …“. I suggest re-phrasing the section as it is somewhat confusing in the midst of very detailed assumptions.

***We agree that the original wording could be confusing due to the simultaneous presentation of global and taxonomic-level interpretations. To improve clarity, we have revised the paragraph to clearly separate overall community structure from genus-level differences, while reducing speculative language.***

Discussion, total and especially line 468: “While the limited sample size prevents definitive conclusions, our…“ The discussion goes to lengths to analyse specifically each individual bacterium. Then the authors suggest that the sample size prevents conclusions. I recommend re-phrasing the discussion or at least deleting that the study is too small (which is readily quite apparent to the reader).

***We agree that the original phrasing may have overemphasized the limitation of sample size. Our intention was to acknowledge study constraints while maintaining a balanced interpretation of the findings. To address this, we have revised the statement to avoid undermining the strength of the analysis, while still appropriately contextualizing the results. The revised text emphasizes the exploratory nature of the study and the consistency of observed patterns, rather than focusing on limitations.***

7. PLOS authors have the option to publish the peer review history of their article ([what does this mean?](https://journals.plos.org/plosone/s/editorial-and-peer-review-process#loc-peer-review-history)). If published, this will include your full peer review and any attached files.


If you choose “no”, your identity will remain anonymous but your review may still be made public.


**Do you want your identity to be public for this peer review?** For information about this choice, including consent withdrawal, please see our [Privacy Policy](https://www.plos.org/privacy-policy).

Reviewer #1: No

Reviewer #2: No

Reviewer #3: **Yes:** PRINCE ANIM ADDO

Reviewer #4: **Yes:** Dr. med. Patrick Biggar

[NOTE: If reviewer comments were submitted as an attachment file, they will be attached to this email and accessible via the submission site. Please log into your account, locate the manuscript record, and check for the action link "View Attachments". If this link does not appear, there are no attachment files.]

To ensure your figures meet our technical requirements, please review our figure guidelines: https://journals.plos.org/plosone/s/figures

You may also use PLOS’s free figure tool, NAAS, to help you prepare publication quality figures: https://journals.plos.org/plosone/s/figures#loc-tools-for-figure-preparation.

NAAS will assess whether your figures meet our technical requirements by comparing each figure against our figure specifications.
