# Supplementary material for: Breastmilk microbiota and its association with infant iron deficiency anaemia: A 16S rRNA metagenomic study in Peruvian mothers cohort
Source: PLoS One. 2026 Aug 27;21(8):e0352428. doi: 10.1371/journal.pone.0352428 (PMC13521397; doi:10.1371/journal.pone.0352428)
Supplement: Peer review history

# Peer review: Decision Letter 0

**DOI:** 10.1371/journal.pone.0352428.r001

**Authors:** Bijit Biswas

1 Sep 2025

<address>PONE-D-25-28555</address><address>Breastmilk Microbiota and Its Association with Infant Anaemia: A 16S rRNA Metagenomic Study in Peruvian Mothers</address><address>PLOS ONE

Dear Dr. Gonzales,

Thank you for submitting your manuscript to PLOS ONE. After careful consideration, we feel that it has merit but does not fully meet PLOS ONE’s publication criteria as it currently stands. Therefore, we invite you to submit a revised version of the manuscript that addresses the points raised during the review process.

==============================

**ACADEMIC EDITOR:**  Thank you for your submission. While the study addresses a novel and relevant question, the manuscript requires major revision. Key issues include small sample size and limited statistical rigor, insufficient methodological detail, overly descriptive and unclear results, speculative discussion with weak clinical integration, and lack of alignment between aims, findings, and framing. Figures, tables, and language also need simplification and standardization. Please respond point-by-point to all four reviewers.</address><address>==============================

Please submit your revised manuscript by Oct 16 2025 11:59PM. If you will need more time than this to complete your revisions, please reply to this message or contact the journal office at plosone@plos.org. When you're ready to submit your revision, log on to https://www.editorialmanager.com/pone/ and select the 'Submissions Needing Revision' folder to locate your manuscript file.

Please include the following items when submitting your revised manuscript:</address>

- <address>A rebuttal letter that responds to each point raised by the academic editor and reviewer(s). You should upload this letter as a separate file labeled 'Response to Reviewers'.</address>
- <address>A marked-up copy of your manuscript that highlights changes made to the original version. You should upload this as a separate file labeled 'Revised Manuscript with Track Changes'.</address>
- <address>An unmarked version of your revised paper without tracked changes. You should upload this as a separate file labeled 'Manuscript'.</address>

<address>If you would like to make changes to your financial disclosure, please include your updated statement in your cover letter. Guidelines for resubmitting your figure files are available below the reviewer comments at the end of this letter.

If applicable, we recommend that you deposit your laboratory protocols in protocols.io to enhance the reproducibility of your results. Protocols.io assigns your protocol its own identifier (DOI) so that it can be cited independently in the future. For instructions see: https://journals.plos.org/plosone/s/submission-guidelines#loc-laboratory-protocols. Additionally, PLOS ONE offers an option for publishing peer-reviewed Lab Protocol articles, which describe protocols hosted on protocols.io. Read more information on sharing protocols at https://plos.org/protocols?utm_medium=editorial-email&utm_source=authorletters&utm_campaign=protocols.

We look forward to receiving your revised manuscript.</address><address>Kind regards,</address><address>Bijit Biswas, MBBS, MD, DNB

Academic Editor

PLOS ONE</address><address>Journal Requirements:</address><address>

When submitting your revision, we need you to address these additional requirements.

1. Please ensure that your manuscript meets PLOS ONE's style requirements, including those for file naming. The PLOS ONE style templates can be found at

https://journals.plos.org/plosone/s/file?id=wjVg/PLOSOne_formatting_sample_main_body.pdf and

https://journals.plos.org/plosone/s/file?id=ba62/PLOSOne_formatting_sample_title_authors_affiliations.pdf

2. PLOS requires an ORCID iD for the corresponding author in Editorial Manager on papers submitted after December 6th, 2016. Please ensure that you have an ORCID iD and that it is validated in Editorial Manager. To do this, go to ‘Update my Information’ (in the upper left-hand corner of the main menu), and click on the Fetch/Validate link next to the ORCID field. This will take you to the ORCID site and allow you to create a new iD or authenticate a pre-existing iD in Editorial Manager.

3. We note that the grant information you provided in the ‘Funding Information’ and ‘Financial Disclosure’ sections do not match.

When you resubmit, please ensure that you provide the correct grant numbers for the awards you received for your study in the ‘Funding Information’ section.

4. Please amend the manuscript submission data (via Edit Submission) to include author Gonzales-Rodriguez AO

5. Please amend your authorship list in your manuscript file to include author Arturo Gonzales

6. Please amend your list of authors on the manuscript to ensure that each author is linked to an affiliation. Authors’ affiliations should reflect the institution where the work was done (if authors moved subsequently, you can also list the new affiliation stating “current affiliation:….” as necessary).

7. Please upload a new copy of Figures 1 and 2 as the detail is not clear. Please follow the link for more information: https://blogs.plos.org/plos/2019/06/looking-good-tips-for-creating-your-plos-figures-graphics/"" https://blogs.plos.org/plos/2019/06/looking-good-tips-for-creating-your-plos-figures-graphics/

</address><address> </address><address>8. If the reviewer comments include a recommendation to cite specific previously published works, please review and evaluate these publications to determine whether they are relevant and should be cited. There is no requirement to cite these works unless the editor has indicated otherwise.

Additional Editor Comments:

Thank you for your submission. While the study addresses a novel and relevant question, the manuscript requires major revision. Key issues include small sample size and limited statistical rigor, insufficient methodological detail, overly descriptive and unclear results, speculative discussion with weak clinical integration, and lack of alignment between aims, findings, and framing. Figures, tables, and language also need simplification and standardization. Please respond point-by-point to all four reviewers.

[Note: HTML markup is below. Please do not edit.]

Reviewers' comments:

Reviewer's Responses to Questions</address><address>**Comments to the Author**

1. Is the manuscript technically sound, and do the data support the conclusions?

The manuscript must describe a technically sound piece of scientific research with data that supports the conclusions. Experiments must have been conducted rigorously, with appropriate controls, replication, and sample sizes. The conclusions must be drawn appropriately based on the data presented. </address><address>Reviewer #1: Partly</address><address>Reviewer #2: Yes</address><address>Reviewer #3: Partly</address><address>Reviewer #4: No</address>

**********

<address>2. Has the statistical analysis been performed appropriately and rigorously? </address><address>Reviewer #1: Yes</address><address>Reviewer #2: Yes</address><address>Reviewer #3: No</address><address>Reviewer #4: Yes</address>

**********

<address>3. Have the authors made all data underlying the findings in their manuscript fully available?

The PLOS Data policy requires authors to make all data underlying the findings described in their manuscript fully available without restriction, with rare exception (please refer to the Data Availability Statement in the manuscript PDF file). The data should be provided as part of the manuscript or its supporting information, or deposited to a public repository. For example, in addition to summary statistics, the data points behind means, medians and variance measures should be available. If there are restrictions on publicly sharing data—e.g. participant privacy or use of data from a third party—those must be specified.</address><address>Reviewer #1: Yes</address><address>Reviewer #2: Yes</address><address>Reviewer #3: Yes</address><address>Reviewer #4: Yes</address>

**********

<address>4. Is the manuscript presented in an intelligible fashion and written in standard English?

PLOS ONE does not copyedit accepted manuscripts, so the language in submitted articles must be clear, correct, and unambiguous. Any typographical or grammatical errors should be corrected at revision, so please note any specific errors here.</address><address>Reviewer #1: Yes</address><address>Reviewer #2: No</address><address>Reviewer #3: Yes</address><address>Reviewer #4: Yes</address>

**********

<address>5. Review Comments to the Author

Please use the space provided to explain your answers to the questions above. You may also include additional comments for the author, including concerns about dual publication, research ethics, or publication ethics. (Please upload your review as an attachment if it exceeds 20,000 characters)</address><address>Reviewer #1: General comments: Thank you for the opportunity to review this paper. Overall, I recommend the authors review the manuscript for consistency in italicizing bacterial species and family names. Additionally, the discussion section is limited and should be expanded to clearly explain the relevance of the bacterial taxa identified in the IDA and non-IDA groups. This will help strengthen the impact and significance of the study.

Title: “Peruvian mothers” is broad, and implies national-level representation. I suggest you consider …” Peruvian mother cohort”

Abstract: This should be 16S rRNA sequencing.

Line 46-54: Although this briefly mentions anemia rates in Peru, I recommend placing greater emphasis on national and regional anemia statistics, particularly in Piura, the city where the participants were recruited.

Line 50-55: The authors mention that socioeconomic inequalities contribute to anemia but then transition to a discussion on the gut microbiota without clearly explaining the connection. I suggest they explicitly state that differences in socioeconomic status can lead to poor dietary intake, which may in turn affect gut microbiota composition and subsequently influence breast milk microbiota. Additionally, more emphasis should be placed on breast milk microbiota rather than gut microbiota, given the focus of the study.

Line 54: Please indicate which regions in Peru.

Line 55-56, 65-66: The authors state that the gut microbiota plays a crucial role and mention that iron levels can alter the intestinal microbiota in adult women. However, the connection between iron status and gut microbiota is not clearly explained. Also, this paper focuses on breast milk microbiota, not gut microbiota. However, the authors include information about the gut microbiota without clearly linking it to the study's main focus. I recommend placing greater emphasis on breast milk microbiota and its influence on the infant’s gut microbiota, while limiting discussion on the gut microbiota itself unless it is directly relevant and clearly integrated into the study's context.

Line 68-71: This section needs to be more specific. The significance of the microbial groups described here is not explained. What roles do these bacteria play in infant health or development? I recommend the authors elaborate on the functional relevance of these taxa to provide more meaningful context.

Line 72: Please clarify, is it “healthy” or “apparent healthy” women.

Line 81: The manuscript should provide more specific information regarding the health status of the mothers, how was this assessed or defined? Additionally, since anemia is more prevalent in populations with limited access to resources and malnutrition, it is important to clarify whether these factors were considered. Breast milk composition can be influenced by various environmental and lifestyle factors, please specify which factors were controlled for or accounted for in the study.

Line 99-100: Which types of growth and cognitive measures were collected? The current description is too general.

Line 103: Were fecal samples collected from infants? If so, this should be clearly stated here. Also, how long were the samples stored at room temperature? Additionally, it should be clarified whether fecal samples were collected solely for parasite identification. If that is the case, please state this clearly in the methods section.

Line 106: I suggest the authors explain why this method was chosen. Was this method selected for reasons of convenience, accessibility, or another justification? Please clarify in the methods section.

Line 110: Provide references on the method used.

Line 113: More details are needed regarding breast milk collection. Was the sample collected at a specific time of day or stage of feeding? Were participants instructed to maintain their regular diets and routines prior to sample collection? Please clarify these aspects to better understand the consistency and context of the samples.

Line 177-179: Why were these factors chosen for analysis? Please clarify. What about other environmental factors such as maternal diet, socioeconomic status, or lifestyle? These variables are likely more relevant and could have a greater impact on both anemia and breast milk composition. I recommend the authors consider including or discussing these factors.

Line 180: anemia is a repeated word

Line 201: need to correct to “per”

Line 203-204: Based on the WHO reference cited in the paper, this study included children with mild to moderate anemia, please clarify.

Line 210: The term “Blastocystis hominis” needs to be italicized. Additionally, it should be clearly indicated that this organism is a parasite and explain why it was evaluated in the study. Even if this seems obvious to the authors, providing these details will help readers understand its relevance.

Line 211: not clear what (n) means for children with IDA and non-IDA.

Line 215: Change determine to determined

Line 229: Use past tense: “was observed” instead of “is observed”

Line 267: Could you please clarify what you are comparing the significant differences in healthy participants to? The current statement is unclear.

Line 271-274: Please revise the grammar here to make this statement clearer and easier to understand. I suggest: “Further analysis did not identify any bacterial genus correlated with hemoglobin concentration. However, some bacterial populations showed weak positive correlations with demographic characteristics, such as maternal age and gestational age at birth.” Check if this is what you want to express.

Line 287: Streptococcus should be italicized. In general, I recommend the authors review the entire manuscript to ensure that bacterial genera and families are consistently and correctly formatted.

Line 288: Is “Dave” a reference? If so, it needs to be properly indicated.

Line 301-303: please clarify which groups you are referring to.

Line 315: Please specify what IL-1β refers to. While this may be obvious to the authors, I suggest clarifying that it is a pro-inflammatory cytokine. Also, I will please explain the relevance of including this statement in relation to your findings.

Line 326-327: please indicate the role or significance of Muribaculum, Nitrospira, and Paludibacter in breast milk or infant health.

Line 331: This statement is quite general. Please elaborate on which specific pathologies are included under “multiple pathologies” in the non-IDA group.

Line 333-334: Please revise the sentence to accurately reflect the observed differences and help readers understand the reduction you are referring to.

Line 348-355. Even though the authors have clearly stated the limitations of this study, I encourage them to discuss the relevance of their results especially for the population of Piura. For instance, how might these findings inform early detection strategies, nutritional interventions, or maternal health programs in this region? Your study provides an important evidence base that contributes to the scientific understanding of breast milk microbiota in relation to anemia, and has the potential to support and guide future community-based interventions, health policy planning, and targeted research efforts in low-resource settings.

Line 354: change “specie” to “species”

Line 355: The statement regarding the Streptococcus genus is too general. Please clarify the relevance of this genus in the context of your study, why is its presence or abundance important?

Line 360: Same as comment above, please clarify the relevance of Corynebacterium in IDA.

Conclusion section is missing.</address><address>Reviewer #2: This study investigates the association between breast milk microbiota and anaemia subtypes in infants (IDA and non-IDA), which is a novel and relevant research question with evident public health importance. The use of 16S rRNA sequencing is a strength, and the focus on a vulnerable population makes the work meaningful. The findings on increased microbial richness in non-IDA and the enrichment of Corynebacterium in IDA are interesting and could provide the basis for future hypotheses.

However, the manuscript requires major revision to reach publication quality. The sample size is small (n=46, divided into three groups), and multiple comparisons are reported without clear statistical adjustment, raising concerns about reliability. Applying correction for multiple testing and reporting effect sizes would strengthen confidence in the results. The Results section is overly descriptive; the text should focus on the main findings, with detailed genus-level data moved to supplementary material. Figures require simplification, correction of typographical errors, and a more explicit focus on biologically relevant taxa. The Discussion is well-referenced but sometimes speculative. For example, the suggestion of compensatory microbiota strategies should be presented as a hypothesis rather than a conclusion. The limitations section is helpful but could be expanded to acknowledge the single time point design, the absence of infant gut microbiota data, potential confounders such as maternal diet or breastfeeding mode, and the relatively small effect sizes seen in beta diversity analysis. In addition, the manuscript requires careful language editing to improve clarity and flow, and the novelty of the work should be emphasised more strongly in the Introduction. Finally, there are inconsistencies in font size across the manuscript (some in 11 pt and others in 12 pt), which should be standardised.</address><address>Reviewer #3: There is great potential in the research work, but it lacks several basic information that needs to be addressed.

1. Based on manual stimulation of skin, did you consider normal flora of skin around the breasts that can be introduced as part of the sample being collected and how was that issue addressed in your methodology?

2. Using the hemoglobinometer for Hemoglobin estimation, what was the safeguard protocol implemented to ensure accurate Hemoglobin estimation if veinous blood sample was collected and assessed using a compact full blood count analyzer.

3. Did you ensure babies were not on any antibiotics before taking stool samples to avoid interference with accurate results.

4. Was the breast milk stored immediately after collection, kept at room temperature for a specified period of time before refrigeration, etc since these factors can affect the microbiota of the breast milk?

5. How was the purity of the extracted DNA samples determined, any immunoblot testing done?

These issues need to be addressed to reflect the scope of work done as extensive and scientifically viable since your sample size is the bare minimum and does not really have a good representation of the health concerns being raised to affect populations in Peru.</address><address>Reviewer #4: To the authors:

Page 1, Abstract, line14 and Page 2, Introduction, line 41: “is estimated 1.8 billion….“. Please define the number: people? Dollars?

Page 2, Abstract, lines 35-36: “… with IDA and non-IDA…“ The whole article revolves around the difference between healthy and IDA infants on the one side and non-IDA infants on the other. Thus, it may be more descriptive to rephrase the title. Furthermore, this would also suggest reviewing the keywords (line 38)as they concentrate on IDA infants.

Page 3, Introduction, line 3: “Children that have suffered anaemia…“ I suggest “Children that suffer from anaemia …

Page 3, Introduction, line 55: “In that respect, the gut microbiota plays a crucial role…“ I suggest “In that respect, the gut microbiota may play a crucial role…“ The whole article builds up tot he conclusion that iron deficiency anaemia is not related microbiota, whereas non-iron deficiency is relatated. The arising question: What is behind “non-iron deficiency anaemia“ ?

Page 3, Introduction, line 60: “ … acids (SCFAs), which contributes to the …“ I suggest ““ … acids (SCFAs), which contribute to the …“

Page 6, Collection of breastmilk, lines 113-114: “After the confirmation that the participant…“ I suggest “After confirmation that the participant…“

Page 9, Ethics, lines 164-168. I suggest moving the paragraph up in the text to page 6 before Laboratory.

Page 10, Demographics, line 182: “ …18 and 39 years, with an mea nage of 27,6.“ I suggest “18 to 30 years, with an mean age of 27.6“

Page 10, Demographics, line 184: “...birth was a significally …“ I suggest “… birth was significally…“

Page 10, Demographics, line 188: The authors refer to “differences observed between the groups“. However, they have not explained to which groups they are refering. The definition follows later on page 11.

Page 11, Demographics, lines 203 to 209: The authors do not state the number of childrenin each group.

Page 12, Microbial richness in Hbm, line 225: “… population, Mean values for healthy participants was …“ I suggest “… population, Mean values for healthy participants were …“

Page 12, Microbial richness in Hbm, line 229: “… High degree of diversity is observed…“ I suggest “… High degree of diversity was observed…“

Page 12, Differences … bacterial genera“, lines 253– 254: A verb is missing in the last sentence. I suggest rephrasing the whole paragraph.

Pages 14 – 18, Discussion: The text presently focusses on the bacterial findings without any connection to possible effects of the bacterial colonisation. The authors start their submission with an explanation why iron defiency is important (lines 55-64). But instead of a clinical translation of their findings, they resort to a basic mathematical listing of bacteria without consequences.

Page 16, Discussion, line 307: “… we also detected a Jeotgalibaca sp…“ I suggest “… we also detected Jeotgalibaca sp…“

In general: What is being subsumated as non-iron deficiency. And where do the authors' readout tie in with the clinical situations and consequences. At present, they are disputing any involvement in microbiota and iron deficiency-- which contradicts the build-up of the introduction.</address>

**********

<address>6. PLOS authors have the option to publish the peer review history of their article (what does this mean?). If published, this will include your full peer review and any attached files.

If you choose “no”, your identity will remain anonymous but your review may still be made public.

**Do you want your identity to be public for this peer review?**  For information about this choice, including consent withdrawal, please see our Privacy Policy.</address><address>Reviewer #1: No</address><address>Reviewer #2: No</address><address>Reviewer #3: **Yes:**  PRINCE ANIM ADDO</address><address>Reviewer #4: **Yes:**  Dr. Patrick Biggar</address>

**********

<address>[NOTE: If reviewer comments were submitted as an attachment file, they will be attached to this email and accessible via the submission site. Please log into your account, locate the manuscript record, and check for the action link "View Attachments". If this link does not appear, there are no attachment files.]

While revising your submission, please upload your figure files to the Preflight Analysis and Conversion Engine (PACE) digital diagnostic tool, https://pacev2.apexcovantage.com/. PACE helps ensure that figures meet PLOS requirements. To use PACE, you must first register as a user. Registration is free. Then, login and navigate to the UPLOAD tab, where you will find detailed instructions on how to use the tool. If you encounter any issues or have any questions when using PACE, please email PLOS at figures@plos.org. Please note that Supporting Information files do not need this step.</address>

Attachment Submitted filename: Plos One Review.pdf

# Author comment: Author response to Decision Letter 1

**DOI:** 10.1371/journal.pone.0352428.r002

26 Jan 2026

Please use the space provided to explain your answers to the questions above. You may also include additional comments for the author, including concerns about dual publication, research ethics, or publication ethics. (Please upload your review as an attachment if it exceeds 20,000 characters)

Reviewer #1: General comments: Thank you for the opportunity to review this paper. Overall, I recommend the authors review the manuscript for consistency in italicizing bacterial species and family names. Additionally, the discussion section is limited and should be expanded to clearly explain the relevance of the bacterial taxa identified in the IDA and non-IDA groups. This will help strengthen the impact and significance of the study.

Title: “Peruvian mothers” is broad, and implies national-level representation. I suggest you consider …” Peruvian mother cohort”

Corrected

Abstract: This should be 16S rRNA sequencing.

Corrected

Line 46-54: Although this briefly mentions anemia rates in Peru, I recommend placing greater emphasis on national and regional anemia statistics, particularly in Piura, the city where the participants were recruited.

Information about Piura was included.

Line 50-55: The authors mention that socioeconomic inequalities contribute to anemia but then transition to a discussion on the gut microbiota without clearly explaining the connection. I suggest they explicitly state that differences in socioeconomic status can lead to poor dietary intake, which may in turn affect gut microbiota composition and subsequently influence breast milk microbiota. Additionally, more emphasis should be placed on breast milk microbiota rather than gut microbiota, given the focus of the study.

Corrected

Line 54: Please indicate which regions in Peru.

Corrected: The case of Piura was specified

Line 55-56, 65-66: The authors state that the gut microbiota plays a crucial role and mention that iron levels can alter the intestinal microbiota in adult women. However, the connection between iron status and gut microbiota is not clearly explained. Also, this paper focuses on breast milk microbiota, not gut microbiota. However, the authors include information about the gut microbiota without clearly linking it to the study's main focus. I recommend placing greater emphasis on breast milk microbiota and its influence on the infant’s gut microbiota, while limiting discussion on the gut microbiota itself unless it is directly relevant and clearly integrated into the study's context.

Corrected: The paragraphs have been revised to more clearly introduce the role of the human breastmilk microbiome

Line 68-71: This section needs to be more specific. The significance of the microbial groups described here is not explained. What roles do these bacteria play in infant health or development? I recommend the authors elaborate on the functional relevance of these taxa to provide more meaningful context.

Corrected: The paragraphs have been revised

Line 72: Please clarify, is it “healthy” or “apparent healthy” women.

Corrected: It was specified

Line 81: The manuscript should provide more specific information regarding the health status of the mothers, how was this assessed or defined? Additionally, since anemia is more prevalent in populations with limited access to resources and malnutrition, it is important to clarify whether these factors were considered. Breast milk composition can be influenced by various environmental and lifestyle factors, please specify which factors were controlled for or accounted for in the study.

According to the manuscript, maternal health status—which determined eligibility for inclusion in the study—was defined based on the following criteria: recent antibiotic use, concurrent chemotherapy, HIV infection, and the presence of mammary gland inflammation. Additionally, socioeconomic homogeneity between study groups was ensured using a modified questionnaire that collected information on housing conditions, energy source, water source, and home ownership status (owned or rented), all of which were comparable across groups, without significant intergroup differences. This information is described in detail in the manuscript.

Line 99-100: Which types of growth and cognitive measures were collected? The current description is too general.

Corrected: The text was clarified, indicating that only information on birth weight and length was collected. This data was obtained from the “Healthy Child Checkup Card,” an official registry of the Ministry of Health.

Line 103: Were fecal samples collected from infants? If so, this should be clearly stated here. Also, how long were the samples stored at room temperature? Additionally, it should be clarified whether fecal samples were collected solely for parasite identification. If that is the case, please state this clearly in the methods section.

Corrected: It was specified that stool samples were collected exclusively from the infants for parasitological analysis, and that they were stored at room temperature.

Line 106: I suggest the authors explain why this method was chosen. Was this method selected for reasons of convenience, accessibility, or another justification? Please clarify in the methods section.

Corrected: The use of a portable hemoglobinometer was selected primarily for practical and logistical reasons, as haemoglobin measurements were conducted directly in the field during community-based visits. This approach ensured immediate assessment of anaemia status in infants, without the need for laboratory infrastructure. We have clarified this rationale in the Methods section.

Line 110: Provide references on the method used.

Corrected

Line 113: More details are needed regarding breast milk collection. Was the sample collected at a specific time of day or stage of feeding? Were participants instructed to maintain their regular diets and routines prior to sample collection? Please clarify these aspects to better understand the consistency and context of the samples.

Corrected: It was specified

Line 177-179: Why were these factors chosen for analysis? Please clarify. What about other environmental factors such as maternal diet, socioeconomic status, or lifestyle? These variables are likely more relevant and could have a greater impact on both anemia and breast milk composition. I recommend the authors consider including or discussing these factors.

Socioeconomic status was assessed in all participants using a modified version of the National Demographic and Family Health Survey (ENDES), and the nutritional status of the infants was also evaluated for the purpose of determining whether these factors could influence anaemia status across study groups. No statistically significant differences were observed between groups for any of these variables. Although the inclusion of maternal dietary assessment would have provided additional insight—particularly in relation to breast milk composition—it was not evaluated in this study. The methodological decision was based on the fact that, in our setting, dietary assessment tools are subject to high variability and may not accurately reflect micronutrient intake, whereas haemoglobin levels provide a more direct and objective indicator of maternal iron status. For this reason, maternal haemoglobin concentration was used as the primary proxy for nutritional status related to anaemia risk. We have incorporated this clarification into the revised manuscript.

Line 180: anemia is a repeated word

Corrected

Line 201: need to correct to “per”

Corrected

Line 203-204: Based on the WHO reference cited in the paper, this study included children with mild to moderate anemia, please clarify.

Corrected

Line 210: The term “Blastocystis hominis” needs to be italicized. Additionally, it should be clearly indicated that this organism is a parasite and explain why it was evaluated in the study. Even if this seems obvious to the authors, providing these details will help readers understand its relevance.

Corrected and expanded

Line 211: not clear what (n) means for children with IDA and non-IDA.

Corrected

Line 215: Change determine to determined

Corrected

Line 229: Use past tense: “was observed” instead of “is observed”

Corrected

Line 267: Could you please clarify what you are comparing the significant differences in healthy participants to? The current statement is unclear.

Corrected

Line 271-274: Please revise the grammar here to make this statement clearer and easier to understand. I suggest: “Further analysis did not identify any bacterial genus correlated with hemoglobin concentration. However, some bacterial populations showed weak positive correlations with demographic characteristics, such as maternal age and gestational age at birth.” Check if this is what you want to express.

Corrected

Line 287: Streptococcus should be italicized. In general, I recommend the authors review the entire manuscript to ensure that bacterial genera and families are consistently and correctly formatted.

Corrected

Line 288: Is “Dave” a reference? If so, it needs to be properly indicated.

Line 301-303: please clarify which groups you are referring to.

Corrected

Line 315: Please specify what IL-1β refers to. While this may be obvious to the authors, I suggest clarifying that it is a pro-inflammatory cytokine. Also, I will please explain the relevance of including this statement in relation to your findings.

Corrected: The discussion has been expanded

Line 326-327: please indicate the role or significance of Muribaculum, Nitrospira, and Paludibacter in breast milk or infant health.

Corrected: The discussion has been expanded

Line 331: This statement is quite general. Please elaborate on which specific pathologies are included under “multiple pathologies” in the non-IDA group.

Corrected: The discussion has been expanded

Line 333-334: Please revise the sentence to accurately reflect the observed differences and help readers understand the reduction you are referring to.

Corrected: The discussion has been expanded

Line 348-355. Even though the authors have clearly stated the limitations of this study, I encourage them to discuss the relevance of their results especially for the population of Piura. For instance, how might these findings inform early detection strategies, nutritional interventions, or maternal health programs in this region? Your study provides an important evidence base that contributes to the scientific understanding of breast milk microbiota in relation to anemia, and has the potential to support and guide future community-based interventions, health policy planning, and targeted research efforts in low-resource settings.

Corrected: The discussion has been expanded

Line 354: change “specie” to “species”

Corrected

Line 355: The statement regarding the Streptococcus genus is too general. Please clarify the relevance of this genus in the context of your study, why is its presence or abundance important?

Corrected: The discussion has been expanded

Line 360: Same as comment above, please clarify the relevance of Corynebacterium in IDA.

Corrected: The discussion has been expanded

Conclusion section is missing.

Corrected

Reviewer #2: This study investigates the association between breast milk microbiota and anaemia subtypes in infants (IDA and non-IDA), which is a novel and relevant research question with evident public health importance. The use of 16S rRNA sequencing is a strength, and the focus on a vulnerable population makes the work meaningful. The findings on increased microbial richness in non-IDA and the enrichment of Corynebacterium in IDA are interesting and could provide the basis for future hypotheses.

However, the manuscript requires major revision to reach publication quality. The sample size is small (n=46, divided into three groups), and multiple comparisons are reported without clear statistical adjustment, raising concerns about reliability. Applying correction for multiple testing and reporting effect sizes would strengthen confidence in the results. The Results section is overly descriptive; the text should focus on the main findings, with detailed genus-level data moved to supplementary material. Figures require simplification, correction of typographical errors, and a more explicit focus on biologically relevant taxa. The Discussion is well-referenced but sometimes speculative. For example, the suggestion of compensatory microbiota strategies should be presented as a hypothesis rather than a conclusion. The limitations section is helpful but could be expanded to acknowledge the single time point design, the absence of infant gut microbiota data, potential confounders such as maternal diet or breastfeeding mode, and the relatively small effect sizes seen in beta diversity analysis. In addition, the manuscript requires careful language editing to improve clarity and flow, and the novelty of the work should be emphasised more strongly in the Introduction. Finally, there are inconsistencies in font size across the manuscript (some in 11 pt and others in 12 pt), which should be standardised.

• Regarding the statistical considerations, we fully acknowledge the concern raised about the absence of correction for multiple comparisons. However, given the exploratory and pilot nature of our study, the limited statistical power due to the sample size, and the hypothesis-generating objectives, we intentionally opted not to apply strict correction methods such as FDR or Bonferroni. Applying such adjustments in this context could lead to an increased risk of type II errors, masking biologically relevant signals that may be valuable for guiding future research. This decision aligns with current recommendations in microbiome statistics, which emphasise prioritising trend-based interpretation over rigid significance thresholds in exploratory datasets (Gloor et al., Front Microbiol, 2017). Similarly, Noyan et al. (Nat Rev Methods Primers, 2022) highlight that in pilot studies of microbiome research, overly conservative statistical corrections can be counterproductive when the primary aim is hypothesis generation rather than confirmatory inference.

• We appreciate the reviewer observation; however, given the exploratory nature of the study and the limited availability of comparable research in this population, we consider it essential to retain genus-level descriptive detail in the Results section. This information is necessary to adequately contextualise the findings and support hypothesis generation, which is a core objective of the study. Therefore, we have preserved this level of detail to maintain scientific interpretability and transparency.

• The discussion has been revised and expanded to enhance its depth and overall contribution

• The limitations section has been revised to explicitly acknowledge the single time-point design, the absence of infant gut microbiota data, the potential influence of confounding factors such as maternal diet and breastfeeding mode, and the relatively small effect sizes observed in beta diversity analysis. These points have now been clearly integrated, reinforcing the exploratory nature of the study and supporting cautious interpretation of the results.

• We have revised and strengthened the introduction, including improvements to formatting and narrative flow, as well as refining the clarity of the discussion sections.

Reviewer #3: There is great potential in the research work, but it lacks several basic information that needs to be addressed.

1. Based on manual stimulation of skin, did you consider normal flora of skin around the breasts that can be introduced as part of the sample being collected and how was that issue addressed in your methodology?

Thank you for this important observation. We agree that skin-associated microbiota may contribute to the microbial profile detected in breast milk samples, particularly in genera such as Staphylococcus and Cutibacterium. In our study, milk samples were collected following standard aseptic procedures consistent with protocols commonly used in microbiome research; however, we did not perform specific sampling of the skin surface (e.g., swabbing of the areolar region) for comparison. Consequently, we were unable to differentiate the extent of contrib

Attachment Submitted filename: Respuesta revisores.docx

# Peer review: Decision Letter 1

**DOI:** 10.1371/journal.pone.0352428.r003

**Authors:** Bijit Biswas

4 Mar 2026

Dear Dr. Gonzales,

Thank you for submitting your manuscript to PLOS ONE. After careful consideration, we feel that it has merit but does not fully meet PLOS ONE’s publication criteria as it currently stands. Therefore, we invite you to submit a revised version of the manuscript that addresses the points raised during the review process.

==============================

**ACADEMIC EDITOR:**  The manuscript has improved following revision and addresses an interesting research question. However, minor revisions are still needed. The conclusions should remain cautious and reflect the exploratory nature and small sample size of the study. The Results and Discussion sections could be streamlined to focus on the most relevant findings, and minor language and clarity issues should be corrected.

==============================

Please submit your revised manuscript by Apr 18 2026 11:59PM. If you will need more time than this to complete your revisions, please reply to this message or contact the journal office at plosone@plos.org. When you're ready to submit your revision, log on to https://www.editorialmanager.com/pone/ and select the 'Submissions Needing Revision' folder to locate your manuscript file.

- A letter that responds to each point raised by the academic editor and reviewer(s). You should upload this letter as a separate file labeled 'Response to Reviewers'.
- A marked-up copy of your manuscript that highlights changes made to the original version. You should upload this as a separate file labeled 'Revised Manuscript with Track Changes'.
- An unmarked version of your revised paper without tracked changes. You should upload this as a separate file labeled 'Manuscript'.

If applicable, we recommend that you deposit your laboratory protocols in protocols.io to enhance the reproducibility of your results. Protocols.io assigns your protocol its own identifier (DOI) so that it can be cited independently in the future. For instructions see: https://journals.plos.org/plosone/s/submission-guidelines#loc-laboratory-protocols. Additionally, PLOS ONE offers an option for publishing peer-reviewed Lab Protocol articles, which describe protocols hosted on protocols.io. Read more information on sharing protocols at https://plos.org/protocols?utm_medium=editorial-email&utm_source=authorletters&utm_campaign=protocols.

We look forward to receiving your revised manuscript.

Kind regards,

Bijit Biswas, MBBS, MD, DNB

Academic Editor

PLOS One

Journal Requirements:

If the reviewer comments include a recommendation to cite specific previously published works, please review and evaluate these publications to determine whether they are relevant and should be cited. There is no requirement to cite these works unless the editor has indicated otherwise.

Please review your reference list to ensure that it is complete and correct. If you have cited papers that have been retracted, please include the rationale for doing so in the manuscript text, or remove these references and replace them with relevant current references. Any changes to the reference list should be mentioned in the rebuttal letter that accompanies your revised manuscript. If you need to cite a retracted article, indicate the article’s retracted status in the References list and also include a citation and full reference for the retraction notice.

Additional Editor Comments:

The manuscript has improved following revision and addresses an interesting research question. However, minor revisions are still needed. The conclusions should remain cautious and reflect the exploratory nature and small sample size of the study. The Results and Discussion sections could be streamlined to focus on the most relevant findings, and minor language and clarity issues should be corrected.

[Note: HTML markup is below. Please do not edit.]

Reviewers' comments:

Reviewer's Responses to Questions

**Comments to the Author**

Reviewer #1: All comments have been addressed

Reviewer #2: (No Response)

Reviewer #3: All comments have been addressed

Reviewer #4: (No Response)

**********

2. Is the manuscript technically sound, and do the data support the conclusions?

Reviewer #1: Yes

Reviewer #2: Partly

Reviewer #3: Yes

Reviewer #4: No

**********

3. Has the statistical analysis been performed appropriately and rigorously?

Reviewer #1: Yes

Reviewer #2: Yes

Reviewer #3: Yes

Reviewer #4: Yes

**********

4. Have the authors made all data underlying the findings in their manuscript fully available?

The PLOS Data policy

Reviewer #1: Yes

Reviewer #2: Yes

Reviewer #3: Yes

Reviewer #4: Yes

**********

5. Is the manuscript presented in an intelligible fashion and written in standard English?

Reviewer #1: Yes

Reviewer #2: Yes

Reviewer #3: Yes

Reviewer #4: Yes

**********

Reviewer #1: The authors have implemented relevant revisions that have improved the manuscript. However, the conclusion section should be clearly presented, unless it is considered an optional component under the journal’s formatting guidelines.

Reviewer #2: The authors have addressed the main points raised in the previous round of review, and the manuscript has improved in clarity and focus. The exploratory and pilot nature of the study is now more clearly articulated, and the limitations section has been expanded to acknowledge key constraints, including the small sample size, the single time-point design, and potential confounding factors.

The study is technically sound and addresses a novel and relevant research question regarding the association between breast milk microbiota and anaemia subtypes in infants. The conclusions are generally supported by the data, provided that the findings are consistently interpreted within an exploratory, hypothesis-generating framework.

A small number of minor points should still be addressed before acceptance. Any remaining language suggesting causality or definitive conclusions should be revised to better reflect the exploratory design. Reporting effect sizes, where feasible, would strengthen interpretation by allowing assessment of biological relevance independent of statistical significance. In addition, the Results section could be further streamlined by emphasizing the most biologically relevant taxa, with more detailed genus-level descriptions moved to the Supplementary Material. Figures and formatting should also be carefully reviewed to ensure clarity and consistency.

Overall, only minor revisions are required, and addressing these points will further improve the clarity and scientific presentation of the manuscript.

Reviewer #3: THIS IS A GOOD SCIENTIFIC RESEARCH PROJECT WITH GREAT PERSPECTIVES FOR FURTHER STUDIES ON SIMILAR RESEARCH

Reviewer #4: Abstract, line 19: “46 mother-child pairs were recruited from Talara, a town in the northern coast of Peru…“ I suggest “ 46 mother-child pairs were recruited from Talara, a town on the northern coast of Peru …“

Introduction, line 46: “For several decades, the initiatives to reduce…“ I suggest “ For several decades, initiatives to reduce…“

Introduction, line 68: “ contributes to the degradation and uptake of human milk oligosaccharides (HMOs), through…“ I suggest deleting the comma “contributes to the degradation and uptake of human milk oligosaccharides (HMOs) through….“

Introduction, lines 81/82: “ In this study, we analysed the breastmilk microbiota of apparent healthy women within …“ I suggest “ In this study, we analysed the breastmilk microbiota of apparently healthy women within…“

Population, line 89: “ … of Peru, recruitment was conducted from October 4 to December 20, 2019.“ I suggest replacing the comma with either a semicolon or starting a new sentence, e.g. “ … of Peru. Recruitment was conducted from October 4 to December 20, 2019.“

Population, lines 89/90: “ The inclusion criteria included children between 3 …“ Repetition of inclusion. I suggest “Inclusion criteria comprised children between 3…“ and deleting “The..“

Demographics, line 228: “… mean Ferritin concentration of 3.4 μg/L.“ I suggest “ mean ferritin concentration of 3.4 μg/L.“

Microbial richness in hBM, line 249: “… population, Mean values…“ I suggest “… population, mean values…“

Differences according to anaemia status and 12 most common bacterial genera, line 282/283: “… only Corynebacterium, an aerobic, gram-positive bacilli, yielded …“ Either sigular or plural, but not both. I suggest “ only Corynebacterium, an aerobic, gram-positive bacillus, yielded …“

Discussion, lines 320/321: “ In our study, we detected 213 genera across 46 hBM samples, being Streptococcus and Staphylococcus the most abundant, contributing to…“ I suggest “In our study, we detected 213 genera across 46 hBM samples with Streptococcus and Staphylococcus being the most abundant, contributing to…“

Discussion, lines 375-379: “ Interestingly, during iron deficiency, reduced bioavailability of iron in the oral environment has been associated with increased frequency of Streptococcus spp. [33]. In this context, we hypothesise that the reduced abundance of Streptococcus spp. in hBM from mothers of IDA children may reflect diminished breastfeeding frequency and/or duration, …“ These sentences are contradictory: first, iron deficiency has been associated with increased Strept. spp. Then second, reduced strept. spp. may reflect IDA. Please explain.

Furthermore, “we hypothesise“ should read “we hypothesised“.

Also, the statement “… may reflect diminished breastfeeding frequency and/or duration,“ has the potential of destroying this complete paper.

Discussion, total and especially line 262: “… Although no clear separation in overall microbial structure was observed between study groups, differences …“. I suggest re-phrasing the section as it is somewhat confusing in the midst of very detailed assumptions.

Discussion, total and especially line 468: “While the limited sample size prevents definitive conclusions, our…“ The discussion goes to lengths to analyse specifically each individual bacterium. Then the authors suggest that the sample size prevents conclusions. I recommend re-phrasing the discussion or at least deleting that the study is too small (which is readily quite apparent to the reader).

**********

what does this mean?). If published, this will include your full peer review and any attached files.

If you choose “no”, your identity will remain anonymous but your review may still be made public.

**Do you want your identity to be public for this peer review?**  For information about this choice, including consent withdrawal, please see our Privacy Policy

Reviewer #1: No

Reviewer #2: No

Reviewer #3: **Yes:**  PRINCE ANIM ADDO

Reviewer #4: **Yes:**  Dr. med. Patrick Biggar

**********

[NOTE: If reviewer comments were submitted as an attachment file, they will be attached to this email and accessible via the submission site. Please log into your account, locate the manuscript record, and check for the action link "View Attachments". If this link does not appear, there are no attachment files.]

To ensure your figures meet our technical requirements, please review our figure guidelines: https://journals.plos.org/plosone/s/figures

You may also use PLOS’s free figure tool, NAAS, to help you prepare publication quality figures: https://journals.plos.org/plosone/s/figures#loc-tools-for-figure-preparation.

NAAS will assess whether your figures meet our technical requirements by comparing each figure against our figure specifications.

# Author comment: Author response to Decision Letter 2

**DOI:** 10.1371/journal.pone.0352428.r004

2 May 2026

6. Review Comments to the Author

Please use the space provided to explain your answers to the questions above. You may also include additional comments for the author, including concerns about dual publication, research ethics, or publication ethics. (Please upload your review as an attachment if it exceeds 20,000 characters)

Reviewer #1: The authors have implemented relevant revisions that have improved the manuscript. However, the conclusion section should be clearly presented, unless it is considered an optional component under the journal’s formatting guidelines.

We thank the reviewer for this helpful suggestion. Although PLOS ONE does not require a separate conclusion section, we agree that a clearly defined closing statement improves the clarity of the manuscript. We have therefore added a dedicated “Conclusions” section summarizing the main findings and their implications.

Reviewer #2: The authors have addressed the main points raised in the previous round of review, and the manuscript has improved in clarity and focus. The exploratory and pilot nature of the study is now more clearly articulated, and the limitations section has been expanded to acknowledge key constraints, including the small sample size, the single time-point design, and potential confounding factors.

The study is technically sound and addresses a novel and relevant research question regarding the association between breast milk microbiota and anaemia subtypes in infants. The conclusions are generally supported by the data, provided that the findings are consistently interpreted within an exploratory, hypothesis-generating framework.

A small number of minor points should still be addressed before acceptance. Any remaining language suggesting causality or definitive conclusions should be revised to better reflect the exploratory design. Reporting effect sizes, where feasible, would strengthen interpretation by allowing assessment of biological relevance independent of statistical significance. In addition, the Results section could be further streamlined by emphasizing the most biologically relevant taxa, with more detailed genus-level descriptions moved to the Supplementary Material. Figures and formatting should also be carefully reviewed to ensure clarity and consistency.

Overall, only minor revisions are required, and addressing these points will further improve the clarity and scientific presentation of the manuscript.

We thank the reviewer for these constructive and insightful comments. First, we have carefully revised the manuscript to ensure that all language suggesting causality has been replaced with terminology more consistent with the exploratory nature of the study. Expressions implying definitive conclusions have been modified to reflect association-based interpretations.bSecond, we have strengthened the interpretation of our findings by incorporating effect size considerations where appropriate, including measures of variance explained in beta diversity analyses and clearer reporting of relative abundance differences. Third, the Results and Discussion section has been streamlined to emphasize the most biologically relevant taxa. Detailed descriptions of additional genera have been moved to the Supplementary Material to improve clarity and readability. Finally, all figures and formatting have been carefully reviewed and revised to ensure consistency, clarity, and alignment with journal standards.

Reviewer #3: THIS IS A GOOD SCIENTIFIC RESEARCH PROJECT WITH GREAT PERSPECTIVES FOR FURTHER STUDIES ON SIMILAR RESEARCH

Reviewer #4: Abstract, line 19: “46 mother-child pairs were recruited from Talara, a town in the northern coast of Peru…“ I suggest “ 46 mother-child pairs were recruited from Talara, a town on the northern coast of Peru …“

Corrected

Introduction, line 46: “For several decades, the initiatives to reduce…“ I suggest “ For several decades, initiatives to reduce…“

Corrected

Introduction, line 68: “ contributes to the degradation and uptake of human milk oligosaccharides (HMOs), through…“ I suggest deleting the comma „contributes to the degradation and uptake of human milk oligosaccharides (HMOs) through….“

Corrected

Introduction, lines 81/82: “ In this study, we analysed the breastmilk microbiota of apparent healthy women within …“ I suggest “ In this study, we analysed the breastmilk microbiota of apparently healthy women within…“

Corrected

Population, line 89: “ … of Peru, recruitment was conducted from October 4 to December 20, 2019.“ I suggest replacing the comma with either a semicolon or starting a new sentence, e.g. “ … of Peru. Recruitment was conducted from October 4 to December 20, 2019.“

Corrected

Population, lines 89/90: “ The inclusion criteria included children between 3 …“ Repetition of inclusion. I suggest “Inclusion criteria comprised children between 3…“ and deleting “The..“

Corrected

Demographics, line 228: “… mean Ferritin concentration of 3.4 μg/L.“ I suggest “ mean ferritin concentration of 3.4 μg/L.“

Corrected

Microbial richness in hBM, line 249: “… population, Mean values…“ I suggest “… population, mean values…“

Corrected

Differences according to anaemia status and 12 most common bacterial genera, line 282/283: “… only Corynebacterium, an aerobic, gram-positive bacilli, yielded …“ Either sigular or plural, but not both. I suggest “ only Corynebacterium, an aerobic, gram-positive bacillus, yielded …“

Corrected

Discussion, lines 320/321: “ In our study, we detected 213 genera across 46 hBM samples, being Streptococcus and Staphylococcus the most abundant, contributing to…“ I suggest „In our study, we detected 213 genera across 46 hBM samples with Streptococcus and Staphylococcus being the most abundant, contributing to…“

Corrected

Discussion, lines 375-379: “ Interestingly, during iron deficiency, reduced bioavailability of iron in the oral environment has been associated with increased frequency of Streptococcus spp. [33]. In this context, we hypothesise that the reduced abundance of Streptococcus spp. in hBM from mothers of IDA children may reflect diminished breastfeeding frequency and/or duration, …“ These sentences are contradictory: first, iron deficiency has been associated with increased Strept. spp. Then second, reduced strept. spp. may reflect IDA. Please explain.

Furthermore, “we hypothesise“ should read “we hypothesised“.

Also, the statement “… may reflect diminished breastfeeding frequency and/or duration,“ has the potential of destroying this complete paper.

We thank the reviewer for this insightful comment. We agree that, as originally phrased, the sentences may appear contradictory and require clarification.

First, the observation that iron deficiency has been associated with an increased abundance of Streptococcus spp. refers specifically to the oral environment, where reduced iron availability may favour the growth of certain bacterial taxa. In contrast, our findings relate to the human breast milk (hBM) microbiota, which represents a distinct ecological niche influenced by multiple factors beyond systemic or local iron status.

Importantly, the presence of Streptococcus spp. in hBM has been consistently attributed to the retrograde transfer of the infant’s oral microbiota during suckling, rather than direct modulation by iron availability within the mammary gland. Therefore, the lower relative abundance observed in hBM from mothers of IDA children does not contradict previous findings, but rather suggests that the efficiency of microbial transfer between the infant oral cavity and breast milk may be altered.

To address the reviewer’s concern, we have revised the text to clarify that our hypothesis does not attribute the observed difference directly to iron deficiency per se, but to potential modulation of microbial transfer dynamics.

Regarding the statement on breastfeeding frequency and duration, we agree that this requires careful wording. Our intention was not to introduce an unsupported causal claim, but to propose a biologically plausible explanation consistent with the known mechanism of retrograde flow. We have therefore softened this statement to reflect its exploratory nature and to avoid overinterpretation.

Finally, we have corrected “we hypothesise” to “we hypothesised” as suggested.

Discussion, total and especially line 262: “… Although no clear separation in overall microbial structure was observed between study groups, differences …“. I suggest re-phrasing the section as it is somewhat confusing in the midst of very detailed assumptions.

We agree that the original wording could be confusing due to the simultaneous presentation of global and taxonomic-level interpretations. To improve clarity, we have revised the paragraph to clearly separate overall community structure from genus-level differences, while reducing speculative language.

Discussion, total and especially line 468: “While the limited sample size prevents definitive conclusions, our…“ The discussion goes to lengths to analyse specifically each individual bacterium. Then the authors suggest that the sample size prevents conclusions. I recommend re-phrasing the discussion or at least deleting that the study is too small (which is readily quite apparent to the reader).

We agree that the original phrasing may have overemphasized the limitation of sample size. Our intention was to acknowledge study constraints while maintaining a balanced interpretation of the findings. To address this, we have revised the statement to avoid undermining the strength of the analysis, while still appropriately contextualizing the results. The revised text emphasizes the exploratory nature of the study and the consistency of observed patterns, rather than focusing on limitations.

________________________________________

7. PLOS authors have the option to publish the peer review history of their article (what does this mean?). If published, this will include your full peer review and any attached files.

If you choose “no”, your identity will remain anonymous but your review may still be made public.

Do you want your identity to be public for this peer review? For information about this choice, including consent withdrawal, please see our Privacy Policy.

Reviewer #1: No

Reviewer #2: No

Reviewer #3: Yes: PRINCE ANIM ADDO

Reviewer #4: Yes: Dr. med. Patrick Biggar

Attachment Submitted filename: Respuesta revisores v2.docx

# Peer review: Decision Letter 2

**DOI:** 10.1371/journal.pone.0352428.r005

**Authors:** Bijit Biswas

24 May 2026

Dear Dr. Gonzales,

Thank you for submitting your manuscript to PLOS ONE. After careful consideration, we feel that it has merit but does not fully meet PLOS ONE’s publication criteria as it currently stands. Therefore, we invite you to submit a revised version of the manuscript that addresses the points raised during the review process.

==============================

**ACADEMIC EDITOR:**  The manuscript addresses an important topic and shows considerable effort. However, clearer structuring of findings across the healthy, IDA, and non-IDA groups, improved figure readability, and a more concise interpretation of microbiota changes are needed to strengthen the manuscript.

==============================

Please submit your revised manuscript by Jul 08 2026 11:59PM. If you will need more time than this to complete your revisions, please reply to this message or contact the journal office at plosone@plos.org. When you're ready to submit your revision, log on to https://www.editorialmanager.com/pone/ and select the 'Submissions Needing Revision' folder to locate your manuscript file.

- A letter that responds to each point raised by the academic editor and reviewer(s). You should upload this letter as a separate file labeled 'Response to Reviewers'.
- A marked-up copy of your manuscript that highlights changes made to the original version. You should upload this as a separate file labeled 'Revised Manuscript with Track Changes'.
- An unmarked version of your revised paper without tracked changes. You should upload this as a separate file labeled 'Manuscript'.

If you would like to make changes to your financial disclosure, please include your updated statement in your cover letter. Guidelines for resubmitting your figure files are available below the reviewer comments at the end of this letter.

If applicable, we recommend that you deposit your laboratory protocols in protocols.io to enhance the reproducibility of your results. Protocols.io assigns your protocol its own identifier (DOI) so that it can be cited independently in the future. For instructions see: https://journals.plos.org/plosone/s/submission-guidelines#loc-laboratory-protocols. Additionally, PLOS ONE offers an option for publishing peer-reviewed Lab Protocol articles, which describe protocols hosted on protocols.io. Read more information on sharing protocols at https://plos.org/protocols?utm_medium=editorial-email&utm_source=authorletters&utm_campaign=protocols.

As the corresponding author, your ORCID iD is verified in the submission system and will appear in the published article. PLOS supports the use of ORCID, and we encourage all coauthors to register for an ORCID iD and use it as well. Please encourage your coauthors to verify their ORCID iD within the submission system before final acceptance, as unverified ORCID iDs will not appear in the published article. *Only*  the individual author can complete the verification step; PLOS staff *cannot*  verify ORCID iDs on behalf of authors.

We look forward to receiving your revised manuscript.

Kind regards,

Bijit Biswas, MBBS, MD, DNB

Academic Editor

PLOS One

**Journal Requirements:**

1. If the reviewer comments include a recommendation to cite specific previously published works, please review and evaluate these publications to determine whether they are relevant and should be cited. There is no requirement to cite these works unless the editor has indicated otherwise.

2. Please review your reference list to ensure that it is complete and correct. If you have cited papers that have been retracted, please include the rationale for doing so in the manuscript text, or remove these references and replace them with relevant current references. Any changes to the reference list should be mentioned in the rebuttal letter that accompanies your revised manuscript. If you need to cite a retracted article, indicate the article’s retracted status in the References list and also include a citation and full reference for the retraction notice.

**Additional Editor Comments:**

The manuscript addresses an important topic and shows considerable effort. However, clearer structuring of findings across the healthy, IDA, and non-IDA groups, improved figure readability, and a more concise interpretation of microbiota changes are needed to strengthen the manuscript.

[Note: HTML markup is below. Please do not edit.]

Reviewers' comments:

Reviewer's Responses to Questions

**Comments to the Author**

Reviewer #2: All comments have been addressed

Reviewer #4: (No Response)

**********

2. Is the manuscript technically sound, and do the data support the conclusions?

Reviewer #2: Yes

Reviewer #4: Partly

**********

3. Has the statistical analysis been performed appropriately and rigorously?

Reviewer #2: Yes

Reviewer #4: Yes

**********

4. Have the authors made all data underlying the findings in their manuscript fully available?

The PLOS Data policy

Reviewer #2: Yes

Reviewer #4: Yes

**********

5. Is the manuscript presented in an intelligible fashion and written in standard English?

Reviewer #2: Yes

Reviewer #4: Yes

**********

Reviewer #2: The authors have responded well to the previous comments, and the manuscript is now clearer and more balanced in its interpretation. The exploratory nature of the study is appropriately emphasized, and the presentation of the results has improved. Overall, I have no further major concerns and believe the manuscript is suitable for publication.

Reviewer #4: I acknowledge that the authors have invested a huge amount of work in this project.

However, I still have a few remarks.

1) Lines 165-170, Bioformatics analysis: The authors use an abbreviation not explained -- "OTU". I suggest explaining the abbreviation.

2) The title is appropriate that breastmilk microbiota is associated with infant anaemia. But this is where the follwing text becomes confusing for me. The authors have defined 3 groups: healthy, IDA and non-IDA. The authors conclude that IDA showed significant increased corynebacteria and reduced streptococci frequencies, i.e. they are stressing the difference in regard to iron deficiency. However, they repeatedly report throughout the text that bacterial changes were noted to a lesser extent between IDA and healthy participants but more between the IDA plus healthy groups versus non-IDA. It may help if the authors were to structure their results and discussion according to the 3 groups they formed. At the moment, the text is very specialised discussing each bacterium.

3) I cannot read the figures and legends and the print out is also unclear. They are too small and too blurred.

**********

what does this mean?). If published, this will include your full peer review and any attached files.

If you choose “no”, your identity will remain anonymous but your review may still be made public.

**Do you want your identity to be public for this peer review?**  For information about this choice, including consent withdrawal, please see our Privacy Policy

Reviewer #2: No

Reviewer #4: No

**********

[NOTE: If reviewer comments were submitted as an attachment file, they will be attached to this email and accessible via the submission site. Please log into your account, locate the manuscript record, and check for the action link "View Attachments". If this link does not appear, there are no attachment files.]

To ensure your figures meet our technical requirements, please review our figure guidelines: https://journals.plos.org/plosone/s/figures

You may also use PLOS’s free figure tool, NAAS, to help you prepare publication quality figures: https://journals.plos.org/plosone/s/figures#loc-tools-for-figure-preparation.

NAAS will assess whether your figures meet our technical requirements by comparing each figure against our figure specifications.

# Author comment: Author response to Decision Letter 3

**DOI:** 10.1371/journal.pone.0352428.r006

26 May 2026

Additional Editor Comments:

The manuscript addresses an important topic and shows considerable effort. However, clearer structuring of findings across the healthy, IDA, and non-IDA groups, improved figure readability, and a more concise interpretation of microbiota changes are needed to strengthen the manuscript.

We appreciate the editor’s suggestions and have revised the manuscript accordingly.

1. Clearer structuring of findings

The Results and Discussion sections were reorganized to more clearly differentiate findings among healthy, IDA and non-IDA groups. Additional text was added to explain the role of the comparison groups and to maintain the focus on the primary objective of investigating associations between hBM microbiota and IDA.

2. Improved figure readability

All figures were regenerated at higher resolution and exported using publication-quality settings. Font sizes, labels and legends were enlarged to improve readability both on screen and in print.

3. More concise interpretation of microbiota changes

Several discussion sections were shortened and revised to reduce speculative interpretation. Conclusions were also rewritten to better reflect the exploratory nature of the study, emphasizing associations rather than causal relationships.

We believe these modifications have substantially strengthened the manuscript and improved the clarity and interpretation of the findings.

All figures were regenerated and reformatted to improve readability. Font sizes, labels, legends and graphical elements were enlarged, and higher-resolution figure files were prepared to ensure adequate visualization both on screen and in printed format. Figure legends were also revised to improve clarity and facilitate interpretation.

[Note: HTML markup is below. Please do not edit.]

Reviewers' comments:

Reviewer's Responses to Questions

Comments to the Author

1. If the authors have adequately addressed your comments raised in a previous round of review and you feel that this manuscript is now acceptable for publication, you may indicate that here to bypass the “Comments to the Author” section, enter your conflict of interest statement in the “Confidential to Editor” section, and submit your "Accept" recommendation.

Reviewer #2: All comments have been addressed

Reviewer #4: (No Response)

________________________________________

2. Is the manuscript technically sound, and do the data support the conclusions?

The manuscript must describe a technically sound piece of scientific research with data that supports the conclusions. Experiments must have been conducted rigorously, with appropriate controls, replication, and sample sizes. The conclusions must be drawn appropriately based on the data presented.

Reviewer #2: Yes

Reviewer #4: Partly

________________________________________

3. Has the statistical analysis been performed appropriately and rigorously?

Reviewer #2: Yes

Reviewer #4: Yes

________________________________________

4. Have the authors made all data underlying the findings in their manuscript fully available?

The PLOS Data policy requires authors to make all data underlying the findings described in their manuscript fully available without restriction, with rare exception (please refer to the Data Availability Statement in the manuscript PDF file). The data should be provided as part of the manuscript or its supporting information, or deposited to a public repository. For example, in addition to summary statistics, the data points behind means, medians and variance measures should be available. If there are restrictions on publicly sharing data—e.g. participant privacy or use of data from a third party—those must be specified.

Reviewer #2: Yes

Reviewer #4: Yes

________________________________________

5. Is the manuscript presented in an intelligible fashion and written in standard English?

PLOS ONE does not copyedit accepted manuscripts, so the language in submitted articles must be clear, correct, and unambiguous. Any typographical or grammatical errors should be corrected at revision, so please note any specific errors here.

Reviewer #2: Yes

Reviewer #4: Yes

________________________________________

6. Review Comments to the Author

Please use the space provided to explain your answers to the questions above. You may also include additional comments for the author, including concerns about dual publication, research ethics, or publication ethics. (Please upload your review as an attachment if it exceeds 20,000 characters)

Reviewer #2: The authors have responded well to the previous comments, and the manuscript is now clearer and more balanced in its interpretation. The exploratory nature of the study is appropriately emphasized, and the presentation of the results has improved. Overall, I have no further major concerns and believe the manuscript is suitable for publication.

Reviewer #4: I acknowledge that the authors have invested a huge amount of work in this project.

However, I still have a few remarks.

1) Lines 165-170, Bioformatics analysis: The authors use an abbreviation not explained -- "OTU". I suggest explaining the abbreviation.

Corrected

2) The title is appropriate that breastmilk microbiota is associated with infant anaemia. But this is where the follwing text becomes confusing for me. The authors have defined 3 groups: healthy, IDA and non-IDA. The authors conclude that IDA showed significant increased corynebacteria and reduced streptococci frequencies, i.e. they are stressing the difference in regard to iron deficiency. However, they repeatedly report throughout the text that bacterial changes were noted to a lesser extent between IDA and healthy participants but more between the IDA plus healthy groups versus non-IDA. It may help if the authors were to structure their results and discussion according to the 3 groups they formed. At the moment, the text is very specialised discussing each bacterium.

We thank the reviewer for this important observation. We agree that the previous version of the manuscript did not sufficiently explain the rationale for including the non-IDA group and may have inadvertently shifted attention away from the primary objective of the study.

To address this concern, we have substantially revised the Introduction, Methods, Results and Discussion sections.

First, we explicitly clarified that the primary objective of the study was to investigate potential associations between human breast milk (hBM) microbiota and iron deficiency anaemia (IDA), while the non-IDA and healthy groups were included as comparison groups to distinguish microbiota patterns potentially associated with iron deficiency from those associated with anaemia more broadly.

Accordingly:

• The Introduction now explains the rationale for including healthy and non-IDA comparison groups.

• The Methods section explicitly states that the non-IDA and healthy groups were incorporated to facilitate identification of microbiota characteristics potentially associated with iron deficiency rather than anaemia status alone.

• The Results section was reorganized to clearly distinguish findings observed between IDA and healthy participants, IDA and non-IDA participants, and healthy and non-IDA participants.

• The Discussion was substantially restructured to emphasize the primary IDA-related findings while still acknowledging the broader taxonomic variation observed in the non-IDA group.

• Additional text was incorporated to explain that non-IDA represents a heterogeneous category and that interpretation of those findings should therefore be made with caution.

Furthermore, we reduced speculative interpretations and focused the discussion on the principal findings directly related to IDA, namely the increased abundance of Corynebacterium, the lower abundance trend observed for Streptococcus, and the overall modest but measurable microbiota differences associated with iron deficiency anaemia. We believe these revisions have substantially improved the clarity of the manuscript and better align the interpretation of the findings with the original study objective.

3) I cannot read the figures and legends and the print out is also unclear. They are too small and too blurred.

All figures were regenerated and reformatted to improve readability. Font sizes, labels, legends and graphical elements were enlarged, and higher-resolution figure files were prepared to ensure adequate visualization both on screen and in printed format. Figure legends were also revised to improve clarity and facilitate interpretation.

Attachment Submitted filename: Respuesta revisores V3.docx

# Peer review: Decision Letter 3

**DOI:** 10.1371/journal.pone.0352428.r007

**Authors:** Bijit Biswas

11 Jun 2026

Breastmilk Microbiota and Its Association with Infant Iron Deficiency Anaemia: A 16S rRNA Metagenomic Study in Peruvian Mothers cohorts

PONE-D-25-28555R3

Dear Dr. Arturo Gonzales,

We’re pleased to inform you that your manuscript has been judged scientifically suitable for publication and will be formally accepted for publication once it meets all outstanding technical requirements.

Within one week, you’ll receive an e-mail detailing the required amendments. When these have been addressed, you’ll receive a formal acceptance letter and your manuscript will be scheduled for publication.

An invoice will be generated when your article is formally accepted. Please note, if your institution has a publishing partnership with PLOS and your article meets the relevant criteria, all or part of your publication costs will be covered. Please make sure your user information is up-to-date by logging into Editorial Manager at Editorial Manager® and clicking the ‘Update My Information' link at the top of the page. For questions related to billing, please contact billing support.

If your institution or institutions have a press office, please notify them about your upcoming paper to help maximize its impact. If they’ll be preparing press materials, please inform our press team as soon as possible -- no later than 48 hours after receiving the formal acceptance. Your manuscript will remain under strict press embargo until 2 pm Eastern Time on the date of publication. For more information, please contact onepress@plos.org.

Kind regards,

Bijit Biswas, MBBS, MD, DNB

Academic Editor

PLOS One

Additional Editor Comments (optional):

Thank you for carefully addressing the reviewers' comments. The manuscript has improved substantially, and the additional revisions have clarified the exploratory nature of the study and strengthened the interpretation of the findings. The reviewers' concerns have been satisfactorily addressed, and no further major issues have been identified. I have no additional comments and consider the manuscript suitable for publication.

# Editor report: Acceptance letter

**DOI:** 10.1371/journal.pone.0352428.r008

**Authors:** Bijit Biswas

PONE-D-25-28555R3

PLOS One

Dear Dr. Gonzales,

I'm pleased to inform you that your manuscript has been deemed suitable for publication in PLOS One. Congratulations! Your manuscript is now being handed over to our production team.

At this stage, our production department will prepare your paper for publication. This includes ensuring the following:

* All references, tables, and figures are properly cited

* All relevant supporting information is included in the manuscript submission,

* There are no issues that prevent the paper from being properly typeset

You will receive further instructions from the production team, including instructions on how to review your proof when it is ready. Please keep in mind that we are working through a large volume of accepted articles, so please give us a few days to review your paper and let you know the next and final steps.

Lastly, if your institution or institutions have a press office, please let them know about your upcoming paper now to help maximize its impact. If they'll be preparing press materials, please inform our press team within the next 48 hours. Your manuscript will remain under strict press embargo until 2 pm Eastern Time on the date of publication. For more information, please contact onepress@plos.org.

You will receive an invoice from PLOS for your publication fee after your manuscript has reached the completed accept phase. If you receive an email requesting payment before acceptance or for any other service, this may be a phishing scheme. Learn how to identify phishing emails and protect your accounts at https://explore.plos.org/phishing.

If we can help with anything else, please email us at customercare@plos.org.

Thank you for submitting your work to PLOS One and supporting open access.

Kind regards,

PLOS ONE Editorial Office Staff

on behalf of

Dr. Bijit Biswas

Academic Editor

PLOS One
